# High‐Strength, Thermally Stable, and Processable Wood Fiber/Polyamide Composites for Engineering Structural Components

**DOI:** 10.1002/advs.202408708

**Published:** 2024-11-11

**Authors:** Zhengtong You, Haigang Wang, Feng Zhang, Haoyuan Zhang, Chuwen Zou, Zhifang Zhou, Yonggui Wang, Zefang Xiao, Daxin Liang, Qingwen Wang, Wentao Gan, Yanjun Xie

**Affiliations:** ^1^ Key Laboratory of Bio‐based Material Science & Technology (Ministry of Education) Northeast Forestry University Harbin 150040 P. R. China; ^2^ School of Architecture and Civil engineering Heilongjiang University of Science and Technology Harbin 150027 P. R. China; ^3^ Country College of Materials and Energy South China Agricultural University Guangzhou 510642 P. R. China

**Keywords:** interfacial bonding, mechanical strengthening, polymer‐matrix composites, thermal stability, wood fibers

## Abstract

Hybrid wood fiber/plastic composites offer a high‐value‐added utilization for agroforestry waste, which also providing a promising solution for reducing white pollution. However, the interface incompatibility between natural wood fibers and polymers significantly impairs the mechanical properties of the composites. Herein, a straightforward procedure is proposed to solve this problem, involving the removal of low‐thermal‐stability hemicellulose from wood fibers by hydrothermal pretreatment, followed by compositing with polyamide to produce hydrothermally treated wood fiber/polyamide composites (HWPACs). No chemical additives are required to improve the interface compatibility of composites, which simplifies the manufacturing process and provides environmental benefits. The effective removal of hemicellulose (78.35%) significantly increases the onset thermal degradation temperature of hydrothermally treated wood fibers (HWFs) by 27.49 °C. This prevents the generation of micro gaps during thermal processing, thereby improving the interfacial bonding strength between HWFs and polyamide. HWPACs exhibit higher mechanical strength (flexural strength 139.45 MPa) and thermal stability while maintaining a low density (1.22 g cm^−3^). Various lightweight, high‐strength, and multi‐shape materials can be prepared by hot pressing, injecting, and printing HWPACs, suggesting their suitability for applications in engineering structural components.

## Graphical abstract

The thermal stability of hydrothermally treated wood fibers (HWFs) is improved by removing hemicellulose in the wood fibers. HWFs and polyamide can be directly compounded to prepare hydrothermally treated wood fiber/polyamide composites (HWPACs) without any chemical additives throughout the process. The prepared composites exhibit lightweight, high‐strength, thermal stability and processability.

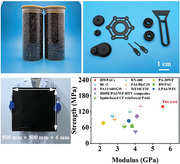

## 1 Introduction

With the rising demand for green consumption and industrial development, high performance engineering structural components are in high demand in various fields such as automobile production, biomedicine, and additive manufacturing. Metal‐based materials (e.g., high‐strength steels and alloys) and petroleum‐based materials (e.g., graphene, carbon fibers, and plastics) with excellent mechanical properties are commonly used to fullfil these requirements in architectural and engineering components.^[^ ^1^, ^2^, ^3^, ^4^ ^]^ However, the high energy consumed in metal processing and the use of unsustainable petroleum‐based materials have negative environmental impacts.^[^ ^5^ ^]^ Moreover, plastic materials remain deficient in strength, and the interface incompatibility between plastic and reinforcement materials constrains their strengthening.^[^ ^6^ ^]^ Therefore, it makes sense to develop alternative materials with easy processing, high‐strength and sustainability.

Wood fibers, a low‐value byproduct of agroforestry, retain the key components of wood (cellulose, hemicellulose, and lignin), thereby offering the advantages of biodegradability and renewability.^[^ ^7^, ^8^, ^9^ ^]^ Compositing wood fibers and plastics can effectively utilize waste resources on a large scale and reduce carbon emissions from petroleum‐based products. However, the poor thermal stability of wood fibers hinders their high‐temperature processing. Hemicellulose is the least thermally stable of the main components in wood cell walls. The thermal degradation of hemicellulose occurs ≈200 °C with the production of pyrolysis gas and bio‐oil.^[^ ^10^ ^]^ The interfacial micro gaps between the wood fibers and the plastic matrix caused by the thermal degradation of wood fibers can deteriorate the interfacial bonding properties of the composites, compromising the reinforcement performance of wood fibers. Therefore, general‐purpose plastics with lower melting points (PE, PP, etc.) are commonly chosen as matrix materials to prevent thermal degradation of wood fibers. Owing to the polarity difference between hydrophilic wood fibers and hydrophobic plastics, chemical additives are required to improve the interfacial bonding properties of the composites. Moreover, the low mechanical strength and heat resistance of general‐purpose plastics hinder their use in high‐performance engineering structural components.^[^ ^11^, ^12^ ^]^

Polyamide possesses excellent mechanical strength and weather resistance compared with common general‐purpose plastics.^[^ ^13^, ^14^ ^]^ Introducing polyamide into wood fiber/plastic composites can effectively improve the mechanical properties of materials and broaden their application areas. In addition, the structure of the amide groups (–CO–NH–) endows polyamide with a chemical polarity similar to that of wood fibers and allows hydrogen bonding with the hydroxyl groups (–OH) in wood fibers.^[^ ^15^ ^]^ However, wood fibers are thermally degraded at polyamide processing temperatures, which severely affects the interfacial bonding and mechanical properties of the composites. Lowering the melting point of polyamide can effectively alleviate the processing problems of wood fiber/polyamide composites. Recently, the melting point of polyamide was successfully reduced through a complex reaction between lithium chloride and polyamide. However, this process disrupts the crystalline structure of polyamide, further impairing its mechanical properties.^[^ ^16^, ^17^ ^]^ Improving the thermal stability of wood fibers is another effective approach to prevent their thermal degradation during processing. Previous studies have demonstrated the improvement of the thermal stability of wood fibers by boric acid treatment. However, this treatment disrupts the crystalline structure of the wood fibers, thereby limiting their reinforcing effect.^[^ ^18^ ^]^ Current solutions to prevent wood fibers thermal degradation during processing commonly require additional chemicals or compromise the mechanical strength of the composites. Therefore, efficiently utilizing wood fibers on a large scale by combining them with polyamide remains a significant challenging.

**Figure 1:**
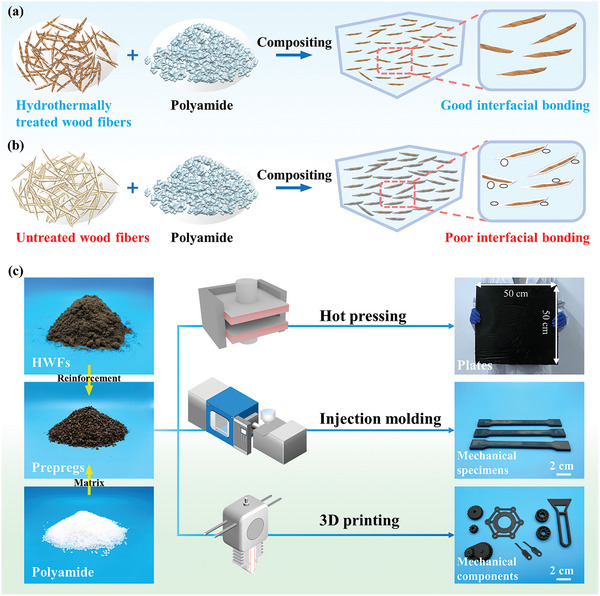
Fabrication and structure of wood fiber/PA6 composites. a,b) Schematic comparison of the compositing processes of HWFs and UWFs with polyamide. c) Processing for HWPACs and photographs of HWPACs materials with different shapes and sizes.

Herein, we employ a straightforward strategy to prepare hydrothermally treated wood fiber/polyamide composites (HWPACs) with high strength, low density, thermal stability, and processability by combining hydrothermal treatment with melt blending. The wood fibers are first hydrothermally pretreated to remove most thermally unstable hemicellulose, resulting in hydrothermally treated wood fibers (HWFs). HWFs exhibit a lower degree of thermal degradation during processing, which significantly facilitates the melt compounding between the HWFs and polyamide. In subsequent molding, HWFs could form tight interactions with polyamide without micro gaps in the composites (**Figure** **1**a). By contrast, thermal degradation of the hemicellulose component creates interfacial micro gaps between the wood fibers and polyamide (Figure 1b), leading to poor interfacial bonding of the untreated wood fiber/polyamide composites (UWPACs). The preparation for HWPACs involves no chemical additives, reducing environmental concerns and costs. The strong interfacial bonding in HWPACs contributes to the efficient stress transfer during the deformation and fracture of the composites. As a result, HWPACs exhibit excellent mechanical properties with a flexural strength of 139.45 MPa and a tensile strength of 76.05 MPa. HWPACs also show significant thermal stability, allowing them to maintain their structural integrity at higher temperatures. Combined with good processability and formability (Figure 1c), HWPACs can be molded in numerous ways to prepare a series of advanced, multi‐shaped, and high‐strength engineering structural components for use in machinery, biomedicine, and other emerging fields.

## 2 Results and Discussion

### 2.1 Microstructure and Thermal Properties of Wood Fibers

**Figure 2:**
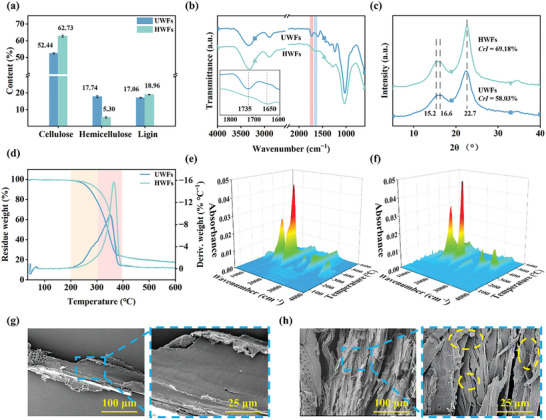
Structure and properties analysis of UWFs and HWFs. a) Composition content. b) FTIR spectrum. c) XRD spectrum. d) TGA and DTG curves. e,f) 3D infrared spectrum of TG–FTIR for UWFs and HWFs, respectively. g,h) SEM images of UWFs and HWFs, respectively.

A straightforward strategy is employed to remove low‐thermal‐stability components from the wood cell walls. The process involved only wood fibers and water, without any additional chemical additives. The hydrothermal treatment yielded a 72.48% solid yield and removed 78.35% of hemicellulose from wood fibers, while slightly affecting the cellulose and lignin content (**Figure** **2**a; Figure S1, Supporting Information). The lower degree of polymerization and branched structure of hemicellulose facilitate hydrolysis during hydrothermal treatment. Initially, the acetyl and glyoxal groups of hemicellulose are shed to form acetic acid. Under acidic conditions, the oligosaccharides and monosaccharides formed by the disruption of glycosidic bonds in hemicellulose are dissolved in a liquid‐phase medium, reducing the hemicellulose content of the wood fibers.^[^ ^19^, ^20^ ^]^ The cellulose‐glycosidic bond is broken slowly under mild acidity during hydrothermal treatment. The occurrence of multiphase reactions is confined to the surface of cellulose due to the intramolecular and intermolecular hydrogen bonding of cellulose. Therefore, hydrothermal treatment causes slight damage to cellulose.^[^ ^21^ ^]^ During hydrothermal treatment, partial lignin is dissolved and removed from the wood cell walls, while residual lignin aggregates and redistributes. Several dissolved lignin forms aggregates and redeposits on wood fibers' surface upon cooling. Therefore, the removal of lignin during hydrothermal treatment is also limited.^[^ ^22^, ^23^ ^]^

The effect of hydrothermal treatment on the functional groups of the wood fibers is reflected in the FTIR spectra (Figure 2b). Distinct characteristic peaks are observed at different wavenumbers in UWFs, including the O─H stretching (3340 cm^−1^) in bonded hydroxyl groups and water, the C–H stretching (2920 and 595 cm^−1^) in aliphatic and aromatic structures, the C ═ O stretching (1735 cm^−1^) in the carbonyl and carboxyl groups in hemicellulose, the C ═ C stretching (1600 cm^−1^) in aromatic rings or double bonds, and the C–O–C stretching (1240 cm^−1^) in ethers.^[^ ^24^ ^]^ In contrast to the UWFs, the peak at 1735 cm^−1^ of the HWFs nearly disappears, indicating that decarbonylation and decarboxylation reactions occur and that hemicellulose in the wood fibers is effectively removed. The C ═ O stretching peak attributed to lignin shifts to a lower wavenumber (1650 cm^−1^), indicating a high lignin content on the surface of the wood fibers after extraction.^[^ ^25^, ^26^ ^]^

Hydrothermal treatment retains the original high‐strength cellulose I crystalline structure of the wood fibers (Figure 2c). The main crystalline peaks in the profiles of UWFs and HWFs are observed ≈2θ = 22.7°, indicative of the cellulose crystallization plane (002). Notably, the strength of the (002) crystal plane increases after the hydrothermal treatment, and the peaks at 2θ = 15.2° and 16.6° corresponding to the (110) and (11_0) crystal planes become evident in the diffraction pattern of HWFs. Both peaks exhibit broad characteristics in the presence of a substantial amount of amorphous material and become clearer as the percentage of the cellulose crystalline region increases.^[^ ^27^, ^28^ ^]^ The effect of hydrothermal treatment on the crystallization behavior of wood fibers was further investigated by calculating the crystallinity using the Segel method.^[^ ^29^ ^]^ The results show a significant increase in the crystallinity of HWFs (69.18%) compared with that of UWFs (58.03%). This improvement can be attributed to the removal of amorphous substances during the hydrothermal treatment, which increases the percentage of cellulose crystalline zones.^[^ ^30^ ^]^

The low thermal stability of wood fibers limits their application in high‐temperature processing. The typical thermal degradation behavior of wood fibers can be observed from the DTG curves of UWFs (Figure 2d). The shoulder peak of the DTG curve at 220–310 °C can be attributed to the thermal degradation of hemicellulose. The low degree of polymerization and amorphous structure of hemicellulose render it susceptible to thermal degradation.^[^ ^10^ ^]^ The peaks in the DTG curve at 310–390 °C are caused by the thermal degradation of cellulose. Compared with hemicellulose, cellulose is more thermally stable due to the presence of strong hydrogen bonds in the crystalline regions, which prevents the rapid destruction of functional groups when heated. With abundant branched chains and aromatic ring structures, lignin exhibits a wide range of chemical bonding activity. Thus, the temperature range in which lignin undergoes thermal degradation is broad and widely distributed.^[^ ^31^, ^32^ ^]^ Compared with UWFs, the final weight loss phase for HWFs occurs from 310 to 390 °C, with only one thermal degradation peak detected owing to the efficient removal of hemicellulose. The onset thermal degradation temperature (*T_o_*) of wood fibers significantly increases from 254.81 °C for UWFs to 282.30 °C for HWFs (Table S1, Supporting Information). Most of the hemicellulose components with low thermal stability have been efficiently removed during the hydrothermal pretreatment, the proportion of cellulose and lignin increases, therefore HWFs exhibit better thermal stability when heated.^[^ ^33^, ^34^ ^]^ The gases released from wood fibers would lead to the micro gaps between the wood fibers and the PA matrix, which directly affects the interfacial bonding and mechanical properties of the composites. Therefore, the pyrolysis gas products were collected and analyzed using thermogravimetry combined with Fourier transform infrared spectroscopy (TG–FTIR). Gaseous products are mainly released from UWFs at 200–400 °C, along with water vapor, methane, carbon dioxide, carbon monoxide, formaldehyde, and methanol (Figure 2e–f; Figure S2, Supporting Information).^[^ ^35^, ^36^ ^]^ Compared with UWFs, HWFs hardly release pyrolysis gases below 300 °C, reducing the impact on the interfacial bonding properties of the composites.

The average size and aspect ratio of HWFs are decreased (Figure S3 and Table S2, Supporting Information), which is attributed to the degradation of unstable hemicellulose and the disruption of wood fiber cell walls during hydrothermal treatment. A notable change is also seen in the color of the wood fibers after hydrothermal treatment. This change is attributed to structural changes in lignin, particularly the increase in the number of chromogenic groups.^[^ ^23^ ^]^ The SEM images of the UWFs show a smooth and flat fiber surface. In contrast, the surface of HWFs after hydrothermal treatment becomes rough and disrupted, forming a porous structure (Figure 2g–h). A few microspheres on the surface of the HWFs are caused by the migratory leaching and cooling deposition of lignin.^[^ ^37^ ^]^ During hydrothermal treatment, the hydrolysis and separation of hemicellulose inside the cell walls, together with the migration and reaggregation of lignin, ultimately lead to the collapse and deformation of the cell walls.^[^ ^38^ ^]^

### 2.2 Properties of the Wood Fiber/PA6 Composites

#### 2.2.1 Interfacial Bonding Properties of As‐Prepared Composites

**Figure 3:**
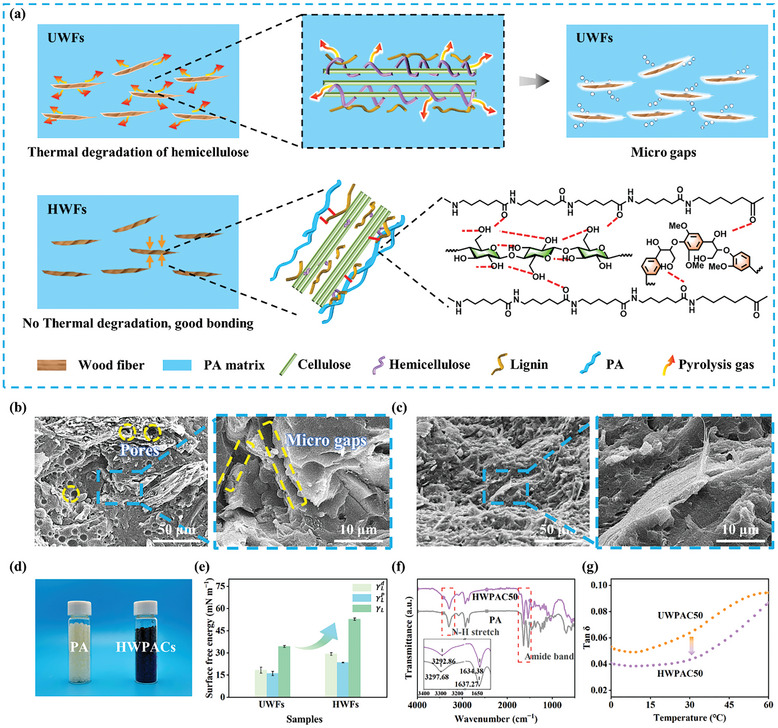
Interfacial bonding properties of as‐prepared composites. a) Schematic comparison of interfacial interaction of UWFs and HWFs with polyamide during processing. b,c) Fracture morphology of UWPACs and HWPACs with 50% wood fiber content, respectively. d) Photographs of polyamide and HWPACs particles. e) Surface energy parameters of UWFs and HWFs. f) FTIR spectrum of polyamide and HWPACs. g) Comparison of loss factors for UWPACs and HWPACs.

The thermal processing temperature of composites (220–250 °C) coincides with the thermal degradation temperature of hemicellulose. The pyrolysis gas from thermal degradation of hemicellulose causes interfacial microgaps to form between UWFs and polyamides (**Figure** **3**a), which hinders the contact between them. In contrast, thermally stable HWFs release fewer pyrolysis gas during processing. The melting polymer matrix could penetrate into the pores of the HWFs. After molding, porous HWFs form strong hydrogen bonds and mechanical interlocking with PA6.^[^ ^39^, ^40^ ^]^ The improved interfacial compatibility is confirmed by SEM images, in which the polyamide matrix shows a smooth fracture surface, while the fracture morphology of the composites becomes significantly rougher with increasing wood fiber content (Figure S4, Supporting Information). The fracture surface between UWFs and PA6 contains numerous micro gaps, indicating that thermal degradation of UWFs affects the interfacial bonding of UWPACs. The HWFs form good interactions with the polyamides through the hydrogen bonding and mechanical interlocking, thus no obvious interfacial micro gaps are observed in the fracture morphology of HWPACs (Figure 3b,c). Wettability, a crucial indicator of interfacial properties, was characterized by contact angle measurements and surface free energy calculations.^[^ ^41^, ^42^ ^]^ The contact angles of HWFs with both water and formamide are lower than that of the UWFs (Figure S5, Supporting Information). Moreover, HWFs exhibit a higher surface free energy (52.51 mN m^−1^) compared with UWFs (34.30 mN m^−1^). The increase in roughness and aliphatic hydroxyl groups in the HWFs lead to a decrease in the contact angle and an increase in the surface wettability.^[^ ^43^ ^]^ This improves the wetting of the HWFs by the molten polyamide and their interfacial bonding. The vibration peaks of the N─H stretching and C ═ O stretching of the PA matrix shift from respectively 3297.68 and 1637.27 cm^−1^ to 3292.86 and 1634.38 cm^−1^, indicating the formation of hydrogen bonds between HWFs and PA (Figure 3f).^[^ ^44^ ^]^ Furthermore, the loss factor was also used to evaluate the interfacial bonding properties of the composites. The *tan δ* values of HWPACs are lower than those of UWPACs during the low‐temperature stage at equivalent wood fiber content (Figure 3g), indicating that composites prepared from HWFs exhibit better interfacial bonding.^[^ ^45^, ^46^ ^]^

#### 2.2.2 Mechanical Properties of As‐Prepared Composites

**Figure 4:**
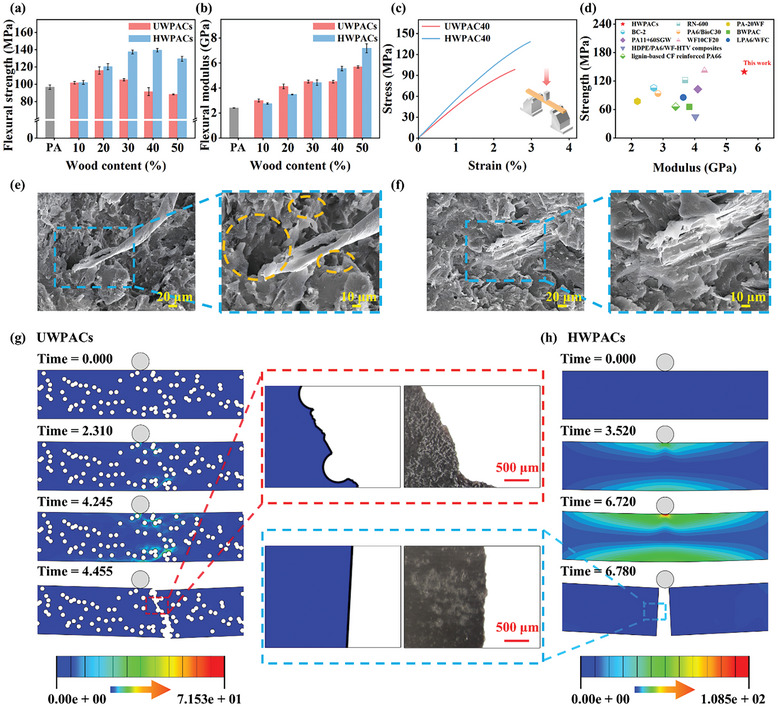
Mechanical properties and mechanical behavior of as‐prepared composites. a,b) flexural strength and flexural modulus of UWPACs and HWPACs with different wood fiber content. c) Flexural stress‐strain curves of typical UWPACs and HWPACs. d) Diagram of the flexural strength‐modulus of HWPACs and reported biomass/polyamide composites (For detailed data see Table S3, Supporting Information). e,f) Flexural damage fractures of UWPACs and HWPACs, respectively. g,h) Stress distribution of UWPACs and HWPACs during three‐point flexural simulation, respectively.

Owing to their good interfacial bonding properties, the HWPACs demonstrate favorable mechanical properties with variations in wood fiber content. The optical flexural strength of HWPACs reaches 139.45 MPa, representing a significant 44.22% increase compared to the flexural strength of PA6 (**Figure** **4**a). As shown in Figure 4d, the flexural strength and modulus of the HWPACs exceed those of natural wood, typical polymers, and numerous reported biomass/polyamide composites, making them competitive for application as engineering structural components. The tensile, flexural, and impact strengths of the HWPACs are higher than those of the UWPACs at equivalent wood fiber content, indicating that hydrothermal treatment improves the mechanical properties of the composites (Figures S7 and S8, Supporting Information). The fracture mechanism of the composites under loaded is further examined through the fracture morphology of UWPACs and HWPACs. An obvious fiber pull‐out phenomenon appears in the flexural and tensile fracture of UWPACs (Figure 4e–f; Figure S9, Supporting Information). This results from the presence of micro gaps between the UWFs and the PA matrix, caused by pyrolysis gases released during the thermal degradation of the UWFs. In contrast, the tight bonding between the HWFs and the polyamide allows the external stresses to be effectively transferred from the matrix to the wood fibers, thereby improving the mechanical strength of the composites.^[^ ^47^ ^]^ In addition, the tight interface effectively dissipates part of the impact energy, which makes the impact toughness of HWPACs better than that of UWPACs (Figure S10, Supporting Information). The mechanical properties of the composites are also affected by changes of wood fiber content. With the increase of wood fiber content, the flexural and tensile strength of the composites first increases and then decreases. The higher proportion of wood fibers as the reinforcing phase enhances the mechanical properties of the composites to withstand greater external loads. However, excessive wood fiber content cause uneven dispersion and agglomerate of fibers in the matrix, leading to stress concentration within the composites. The incorporation of rigid wood fibers limits the mobility of the polyamide molecular chains. Thus, the impact strength of the composites gradually decreases as the wood fiber content increases. However, the creep resistance of the composites can be improved with the increase of wood fibers (Figure S11, Supporting Information). Furthermore, HWPACs exhibit a low density of 1.22 g cm^−3^, which is beneficial for lightweight design and carbon emission reduction. Compared with common metals, plastics, and other reinforcing composites, HWPAC40 features a higher specific flexural strength (Figures S12 and S13, Supporting Information).

The mechanical behavior of fiber reinforced polymer composites under external loads were further studied by finite element analysis.^[^ ^48^, ^49^, ^50^, ^51^, ^52^ ^]^ The micro gaps caused by the thermal degradation of UWFs were determined to critically affect the mechanical properties of the composites through observations above. Therefore, the difference in mechanical behavior between UWPACs and HWPACs was compared by applying pore parts to the UWPACs model. Figure 4g,h illustrate the change in stress distribution during the three‐point flexural simulation. The micro gaps caused by the thermal degradation of wood fibers in the UWPACs function as stress concentration points. These points weaken the structural integrity of the composites, making them more prone to rupture under external stress. The micro gaps also guide the crack extension paths, resulting in irregular and discontinuous fracture paths. In contrast, HWPACs without micro gaps exhibit a more uniform internal structure. The stress applied to the HWPACs is effectively dispersed, resulting in increased damage resistance and good flexural strength.

#### 2.2.3 Dynamic Mechanical and Rheological Properties of As‐Prepared Composites

**Figure 5:**
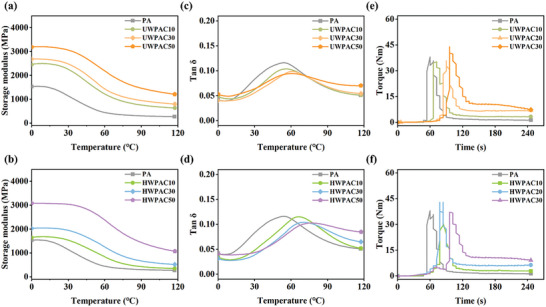
Dynamic mechanical and rheological properties of as‐prepared composites. a,b) Storage modulus curves of the UWPACs and HWPACs, respectively. c,d) Loss factor curves of the UWPACs and HWPACs, respectively. e,f) Torque rheology of the UWPACs and HWPACs, respectively.

The storage modulus corresponding to the material stiffness remains consistent in the low temperature range of 0–30 °C but tends to decrease with increasing temperature (**Figure** **5**a,b), as the molecular chain mobility of PA 6 increases at higher temperatures. Wood fibers are more rigid than polyamide matrix, thus the storage modulus of composites continuously increases with increasing wood fiber content.^[^ ^53^ ^]^ The loss factor is the ratio of the storage modulus to the loss modulus, which is used to evaluate the viscoelastic response of materials. The presence of wood fibers in the composites reduces the *tan δ* peaks (Figure 5c,d), indicating lower internal energy dissipation at the interface between filler and matrix. This reduction can be attributed to the similar chemical polarities of the wood fibers and PA6, leading to excellent interfacial bonding properties of the composites.^[^ ^45^ ^]^ The peaks of the loss factor are used to characterize the glass transition temperatures (*T*_g_) of the composites. The *T*_g_ moves toward high temperatures as the content of wood fiber increases. Wood fibers limit the mobility of the molecular chains in the amorphous region, requiring more heat energy for the glass transition.^[^ ^54^ ^]^ Moreover, the higher *T*_g_ of HWPACs compared to UWPACs at equivalent wood fiber content indicates the stronger interfacial interaction between HWFs and the polyamide matrix. Torque rheology properties are commonly used to characterize the processing properties of polymer‐based composites. The stable torque of the wood fiber/PA6 composites increases with increasing wood fiber content (Figure 5e,f), indicating the increased viscosity and reduced flowability of the composites. This is attributed to the strong interactions between the wood fibers, which increase the stable torque of the composites and decrease the wettability of the polymer matrix to the wood fibers.^[^ ^55^ ^]^ UWPACs and HWPACs, both prepared using only wood fibers and polyamide matrix without lubricants, exhibit similar stable torque at equivalent wood fiber content.

#### 2.2.4 Thermal Properties of As‐Prepared Composites

**Figure 6:**
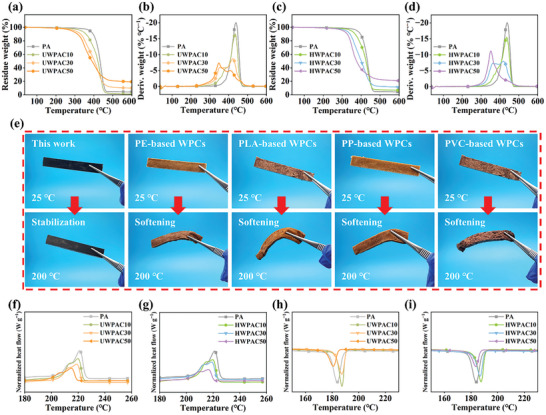
Thermal properties of as‐prepared composites. a,b) and c,d) TGA and DTG curves for UWPACs and HWPACs, respectively. e) Comparison diagram of thermal stability for HWPACs and several common polymer‐based WPCs at 25 and 200 °C. f,h) and g,i) DSC melting and crystallization curves of UWPACs and HWPACs, respectively.

Pure PA6 exhibits a single pyrolysis phase in its DTG curves. However, with the incorporation of wood fibers into the PA6 matrix, the pyrolysis of the composites is categorized into two distinct phases corresponding to the pyrolysis of the wood fibers and PA6 (**Figure** **6**a–d). The maximum degradation rate of the PA6 peak decreases as wood fiber content increases. The residual char layer, generated by the pyrolysis of wood fibers, effectively hinders heat transfer and delays the pyrolysis of PA6.^[^ ^56^ ^]^ The char yield increases with higher wood fiber content (Table S1, Supporting Information), indicating that the wood fibers are the main char‐forming material in the composites.^[^ ^18^ ^]^ Moreover, the incorporation of HWFs improves the thermal stability of HWPACs, as evidenced by their higher *T_o_* and char yield compared with those of UWPACs at equivalent wood fiber content. Thermally stable HWFs effectively constrain the melting deformation of the matrix when heated. Hence, HWPACs can maintain the initial shape during heating from room temperature to 200 °C, whereas other commonly used polymer‐based composites exhibit softening and deformation (Figure 6e). HWPACs also exhibit higher thermal stability compared to the reported biomass/polymer composites (Table S4, Supporting Information), making them potentially valuable for applications in heat‐resistant components.

Both PA6 and the composites exhibit the characteristic melting behavior with two peaks. The main peak corresponds to the α‐crystalline form of PA6, whereas the shoulder peak represents the γ‐crystalline form with lower thermal stability (Figure 6f,g). The melting temperature (*T*_m_) of the polyamide crystals in the composites decreases with an increase in the wood fiber content, indicating a reduction in crystal size as the filler content increases.^[^ ^57^ ^]^ The crystallinity (*X_C_*) of both UWPACs and HWPACs initially increases and then decreases with an increase in the wood fiber content (Table S5, Supporting Information). The increase in *X_C_* can be attributed to the weak nucleating effect of wood fibers.^[^ ^58^ ^]^ However, as the wood fiber content continues to increase, the relative content of the polyamide matrix decreases, and the thermal movement of the molecular chains is restricted, leading to a decrease in *X_C_*.^[^ ^59^ ^]^ The hydrothermal treatment positively affects the crystallinity of the composites. Consistent with other characterization results, HWFs exhibit better interfacial bonding properties with PA6 compared with UWFs, leading to increased interface shear. Therefore, the crystallinity of the HWPACs surpasses that of the UWPACs, which is attributed to the shear effect on the wood fiber surface.^[^ ^60^ ^]^

#### 2.2.5 Multi‐Path Processing and Environmental Impacts of As‐Prepared Composites

**Figure 7:**
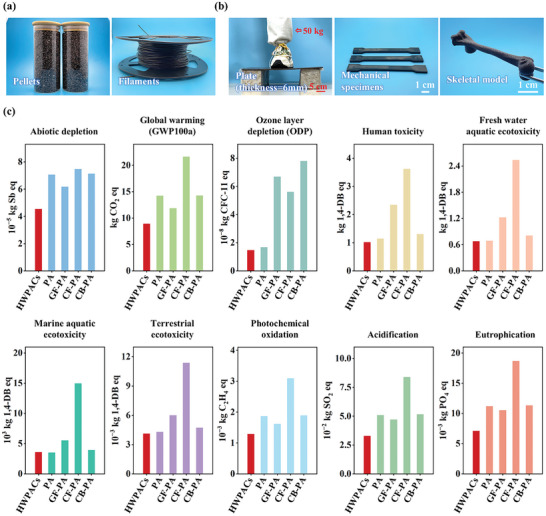
Preparation and environmental impacts of HWPACs. a) Demonstration of HWPACs prepregs with various forms. b) Photographs of multi‐shape HWPACs materials prepared by different processing paths. c) Environmental impacts of preparing HWPACs compared to PA6, GF‐PA, CF‐PA, and CB‐PA per ton.

Wood fibers, a low‐value processing byproduct, are produced in large quantities each year and need to be effectively utilized. The thermal stability of the wood fibers is improved after hydrothermal treatment, which prevents thermal degradation of the wood fibers during processing. Various forms of prepregs can be obtained by direct melt blending of HWFs with PA6 (**Figure** **7**a). The prepared prepregs (pellets and filaments) exhibit good processability and formability, making them suitable for a wide range of processing techniques and multi‐shape development of composites due to the favorable melt flowability and low viscosity of polyamide. Furthermore, the composites prepared through hydrothermal pretreatment show good mechanical properties, and bulk HWPACs‐plates could support a 50 kg adult standing on one foot (Figure 7b).

The development of HWPACs is promising for achieving high value‐added conversion of wood waste through additive‐free treatment. Moreover, the hydrothermal pretreatment shows the advantages of lower processing temperature and simplicity compared to several previous studies, which is conducive to the realization of large‐scale production of polyamide‐based composites.^[^ ^39^, ^61^ ^]^ Additionally, HWPACs can be recycled and reprocessed to reduce resource waste and environmental pollution after their service life (Figure S14, Supporting Information). The mechanical properties of HWPACs continually decrease with increasing number of processing cycles (Figure S15, Supporting Information), because the composites are continuously subjected to heating and shearing during processing.^[^ ^62^, ^63^ ^]^ Remarkably, the mechanical strength of HWPACs remains at ≈70% of the primary composites after four processing cycles. The char yields of HWPACs at equivalent wood fiber content show insignificant differences after various reprocessing cycles. The *T_o_* of HWPACs increases with the number of processing cycles, as the components with low thermal stability degrade during the previous processing (Figure S16, Supporting Information).

HWPACs, prepared by the incorporation of natural wood fibers, potentially offer a lower carbon footprint than pure polyamide products and traditional commercial fiber‐reinforced composites. A cradle‐to‐gate life cycle assessment (LCA) was conducted to quantify the environmental impacts of HWPACs (Figure S17, Supporting Information), polyamide 6 (PA6), glass fiber reinforced polyamide (GF‐PA), carbon fiber reinforced polyamide (CF‐PA), and carbon black reinforced polyamide (CB‐PA) (Figure 7c; Tables S6 and S7, Supporting Information). The results show that the environmental impacts of HWPACs are lower than those of PA6 and commercial traditional fiber reinforced polyamide composites in most categories, suggesting the positive environmental advantages of HWPACs. The main contributors affecting impact categories are the use of polyamide raw materials and energy consumption (Table S8, Supporting Information). Thus, the environmental impacts of HWPACs can be further reduced by decreasing the proportion of polyamide and using energy‐efficient devices and wind power in the current industrial processes. Combined with the abundant raw materials, low carbon emissions, and versatile fabrication options, HWPACs are advantageous and promising materials for engineering structural components in mechanical, biomedical, and other fields.

## 3 Conclusion

In summary, we prepared lightweight hydrothermally treated wood fiber/polyamide composites (HWPACs) with excellent mechanical properties and thermal stability, through a straightforward procedure involving the hydrothermal pretreatment of wood fibers and compositing with polyamide. No chemical additives are required to treat wood fibers and composites, which effectively simplifies the preparation process and brings environmental benefits. The thermal stability of wood fibers is improved by the effective removal of thermally unstable hemicellulose from the cell walls, which reduces the thermal degradation of HWFs in molten polyamide. The high thermal stability of HWFs prevents the generation of interfacial micro gaps during processing, maintaining the structural integrity of the HWPACs, and reducing stress concentrations. Furthermore, the good interfacial bonding allows effective load transfer from matrix to HWFs, resulting in better mechanical properties of HWPACs. The HWPACs exhibit a flexural strength of 139.45 MPa and a tensile strength of 76.05 MPa, representing increases of 44.22% and 23.00% in comparison with polyamide. HWPACs also exhibit high thermal stability compared to other common biomass/polymer hybrid composites, which can maintain the stable initial shape at 200 °C. The production process of HWPACs is simple, and the prepared prepregs are suitable for a variety of processing routes, making them suitable for large‐scale production. Leveraging the abundance and renewability of wood fibers, the high‐performance HWPACs with good interfacial bonding properties and easy processing offer sustainable alternatives for engineering structural components, heat‐resistant devices, and medical instruments.

## 4 Experimental Section

### Materials

Waste poplar chips collected from Harbin were crushed to obtain wood fibers. Polyamide 6 (PA6) particles (1013B) were supplied by UBE Company of Japan with the density of 1.14 g cm^−3^ and melt flow rate of 2.2 g min^−1^ (235 °C, 2.16 kg).

### Hydrothermal Treatment of Wood Fibers

Hydrothermal treatment was conducted in an oil bath cooker (TD1‐Y, Xianyang Tongda Light Industry Equipment Co., Ltd.), which was equipped with eight 1L stainless steel reaction kettles. The absolute dry weight of wood fibers in each kettle was 100 g. The reaction was conducted under the following conditions: solid‐liquid ratio of 1:5, reaction temperature of 180 °C and reaction time of 90 min. After the hydrothermal treatment, the reaction kettles were removed and then cooled to room temperature. The treated wood fibers were washed with water until a neutral state was reached. Both untreated wood fibers (UWFs) and hydrothermally treated wood fibers (HWFs) were dried in an oven at 103 °C for 24 h to achieve a final moisture content of less than 2 wt.%.

### Preparation of Wood Fiber/PA6 Composites

All raw materials were dried at 103 °C for 10 h to remove moisture prior to composites preparation. UWFs and HWFs were thoroughly mixed with polyamide according to the formulation in Table S9 (Supporting Information), respectively. The untreated wood fiber/polyamide composites (UWPACs) and hydrothermally treated wood fiber/polyamide composites (HWPACs) were subsequently prepared by a two‐step process of melt compounding, and mold forming. The mixture was compounded in a twin‐screw plastics extruder (SJSH30, Nanjing Rubber and Plastic Machinery Co., Ltd., Nanjing, China) at 60 rpm. The temperature of the heating zones for the extruder ranged from 200 to 250 °C. The mechanical specimens were injected using an injection molding machine (MA900IIs, China Haitian Plastics Machinery Group Co., Ltd.) with a barrel temperature of 250 °C and an injection and holding pressure of 50 Bar.

### Characterization

Compositional analysis of wood fibers was performed according to the Van Soest analytical method,^[^ ^64^ ^]^ with each specimen test repeated three times. In addition, the yield (*Y*) of HWFs and the removal ratio of each component were calculated using Equations 1 and 2, respectively:

(1) $$Y=\frac{W_{out}}{W_{in}}\;\;\times \;\;100\%$$

(2) $$\mathrm{Removal}\;\mathrm{ratio}\;(\%)=\frac{C_{\mathrm{in}}-C_{\mathrm{out}}*Y}{C_{\mathrm{in}}}\;\;\times \;\;100\%$$

where *W_in_* is the weight of the wood fibers before hydrothermal treatment, *W_out_* is the weight of the hydrothermally treated wood fibers, and *C_in_* and *C_out_* represent the content of each wood fibers component before and after hydrothermal treatment, respectively.

FTIR spectroscopic measurements were performed using a Nicolet 6700 FTIR spectrometer (Thermo Fisher Scientific Co., Ltd., Waltham, MA, United States) in the 4000–600 cm^−1^ range at a scanning rate of 32 scans min^−1^.

Changes in the crystallization behavior of the specimens were analyzed using an X‐ray diffractometer (XRD‐6100, Shimadzu Co., Ltd., Japan) under the conditions of 2θ = 5–40° and scanning rate of 5° min^−1^, with a test voltage of 40 kV and a current of 30 mA. Equation 3 was applied to determine the crystallinity index (*CrI*) of the specimens in accordance with the Segel method:

(3) $$\mathrm{CrI}=\frac{I_{002}-I_{\mathrm{am}}}{I_{002}}\;\;\times \;\;100\%$$

where *CrI* represents the crystallinity index, *I*_002_ denotes the maximum intensity of the diffraction peak of the cellulose 002 lattice (2θ = 22.8°), and *I_am_* is the diffraction intensity of the amorphous peak (2θ = 18.0°) between the 002 and 101 lattice diffraction peaks.

### Morphological Analysis

Optical images of UWFs and HWFs were captured using an ultra‐depth‐of‐field optical microscope (VHX‐S650E, KEYENCE, Japan). The resultant photographs were then analyzed using the software ImageJ. The microscopic morphology and structure of all samples were characterized by scanning electron microscope (Thermo Fisher Scientific, United States) at 12.5 kV.

### Contact‐Angle Measurement and Surface‐Free‐Energy Analysis

Contact angle measurements were conducted using a contact‐angle‐measuring instrument (Attension Theta, Biolin Scientific, Sweden) from 5 µL droplets. The surface free energy of the wood fibers was subsequently calculated according to the Owens–Wendt method,^[^ ^42^ ^]^ and water ($\gamma_{L}^{d}$ = 21.8, $\gamma_{L}^{P}$ = 51.0) and formamide ($\gamma_{L}^{d}$ = 39.0, $\gamma_{L}^{P}$ = 19.0) were used as probe liquids.

### Thermal Analysis

All specimens were dried to absolute dry state before testing. Thermal stability and thermal degradation behavior of the specimens were analyzed by thermogravimetric analyzer (TG209 F1, NETZSCH, Germany). The specimens were heated from room temperature to 600 °C at a rate of 10 °C min^−1^. The pyrolysis gases of the wood fibers were further analyzed using simultaneous thermal analysis–infrared spectrometer (STA 449/F5‐ALPHA). DSC analysis was performed using DSC (Q50, TA, United States) under nitrogen atmosphere. Specimens were heated from room temperature to 260 °C at a heating rate of 20 °C min^−1^ and maintained for 5 min to eliminate heat history. They were then cooled to −30 °C at a rate of 10 °C min^−1^ and reheated to 260 °C at a rate of 10 °C min^−1^. The crystallinity (*X_c_*) of PA6 in the composites was calculated using Equation 4:

(4) $$\mathrm{Xc}=\frac{\Delta H\exp}{\Delta H}\times \frac{1}{W_{f}}\times 100$$

where ΔH_exp_ is the melting enthalpy obtained from experimental testing of PA6, Δ*H* is the melting enthalpy of PA6 when fully crystallized under ideal conditions with an enthalpy value of 240 J g^−1^, and *W_f_* represents the mass fraction of PA6 in the composites.

### Analysis of Mechanical Properties

The flexural properties of the wood fiber/polyamide composites were tested based on ASTM D790 with the test speed of 2 mm min^−1^ and the span of 64 mm. Tensile properties were tested using the ASTM D368 standard at a speed of 5 mm min^−1^for dumbbell‐type specimens. The impact strength was characterized based on the ISO 179 standard, and the tests were conducted in notched impact mode with conditions including 5 J energy level and 2.9 m s^−1^ impact speed.

### Finite Element Analysis

Simulations of three‐point flexural tests of UWPACs and HWPACs were performed by finite element (FE) method using ABAQUS software. The simulations were conducted using a simplified 2D model consisting of a specimen, an indenter and two supports, with the specimen properties being linearly elastic and the indenter and supports set to be rigid materials. The contact constraint between the indenter and the specimen was defined as a hard contact with a friction coefficient in the tangential direction of 0.3. The supports were fixed on both sides of the specimen and limited its movement, and the indenter was pushed downwards with a total displacement of 0.7 mm during the simulation. The HWPACs was set as a homogeneous material while an additional 15% of pores were randomly added to the UWPACs during modelling to investigate the effect of micro gaps inside the composites on the mechanical behavior. The dimensions of both composites were 7 mm x 1 mm, and the fracture was set to a brittle fracture with a strain at break of 0.12.

### Analysis of Dynamic Mechanical Properties and Creep Properties

The dynamic thermomechanical analyzer (Q800, TA, United States) was used to assess the dynamic mechanical properties and creep properties of the composites with the specimen size of 35 mm × 10 mm × 4 mm. The dynamic mechanical properties were tested using a single cantilever fixture in the DMA Multifrequency‐strain module under the following conditions: test amplitude of 50 µm, temperature range of 0–120 °C, and temperature increase rate of 3 °C min^−1^. The samples were loaded with 2 MPa for 30 min and then released from the load for 30 min to measure the creep properties of the composites at 30 °C.

### Analysis of Rheological Properties

The torque rheology curves of the samples were obtained using a torque rheometer (Polylab OS). Dried wood fibers and PA6 were added to the mixing chamber and mixed at 250 °C and 50 rpm for simultaneous torque testing.

### Life‐Cycle Assessment

The LCA analysis was conducted using SimaPro and followed the International Organization for Standardization (ISO) standard series 14 040. The environmental impacts of producing HWPACs per ton compared to polyamide 6 (PA6), glass fiber reinforced polyamide (GF‐PA), carbon fiber reinforced polyamide (CF‐PA), and carbon black reinforced polyamide (CB‐PA) were specifically evaluated.

## Conflict of Interest

The authors declare no conflict of interest.

## Author Contributions

Z.Y. conducted experiments and theoretical analysis. H.W., W.G., and Y.X. conceived the idea and designed the experiments. F.Z. performed the mechanical tests and simulations. H.Z. conducted the LCA. C.Z. and Z.Z. performed the material characterizations. Y.W., Z.X., D.L., and Q.W. supervised experimental design and data interpretation. Z.Y., H.W., W.G., and Y.X. collectively wrote the paper. All authors commented on the final manuscript.

## Supporting information



Supporting Information

## Data Availability

The data that support the findings of this study are available from the corresponding author upon reasonable request.
