# Supplementary material for: High‐Strength, Thermally Stable, and Processable Wood Fiber/Polyamide Composites for Engineering Structural Components
Source: Adv Sci (Weinh). 2024 Nov 11;12(1):2408708. doi: 10.1002/advs.202408708 (PMC11714178; doi:10.1002/advs.202408708)
Supplement: Supplementary file 1 — Supporting Information [file ADVS-12-2408708-s001.docx]

Supporting Information

High-Strength, Thermally Stable, and Formable Wood Fiber/Polyamide Composites for Engineering Structural Components

Zhengtong You, Haigang Wang*, Feng Zhang, Haoyuan Zhang, Chuwen Zou, Zhifang Zhou, Yonggui Wang, Zefang Xiao, Daxin Liang, Qingwen Wang, Wentao Gan*, Yanjun Xie*


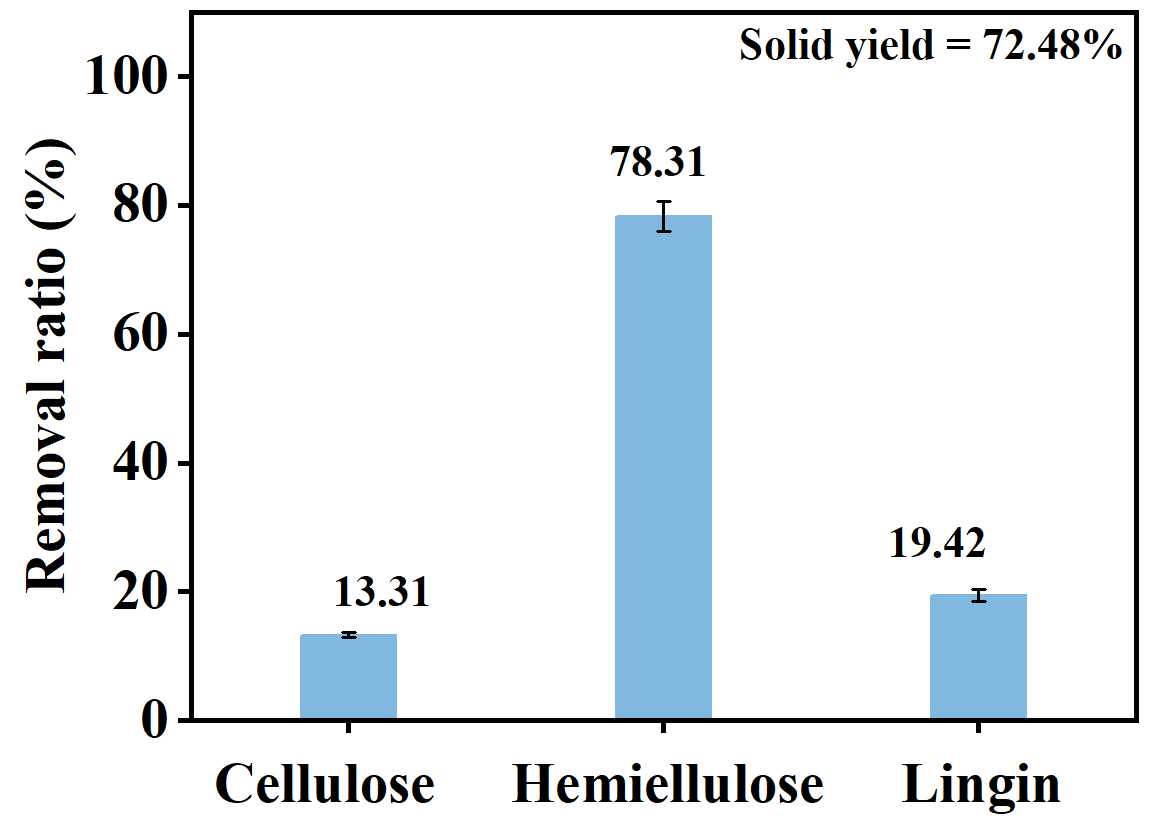


**Figure S1.** Removal ratio of wood fiber components during hydrothermal treatment.


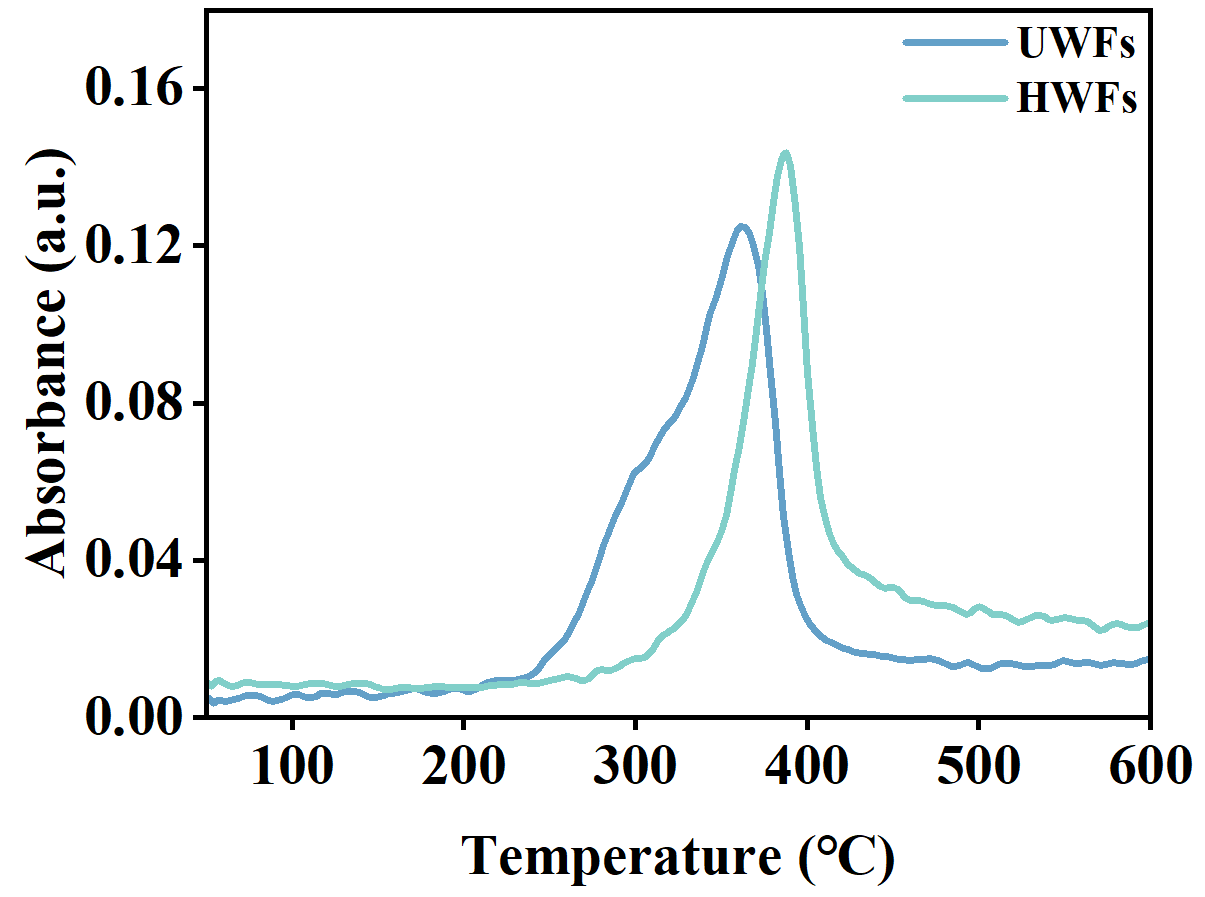


**Figure S2.** Total pyrolysis gas product absorption rate for UWFs and HWFs.


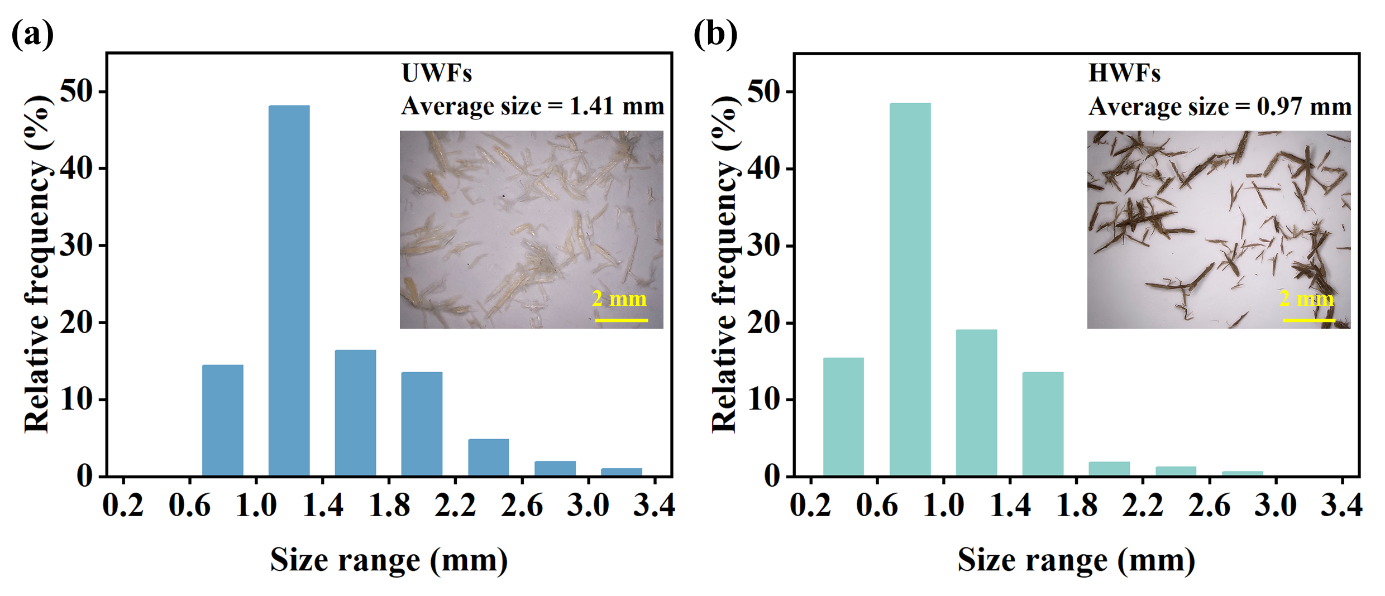


**Figure S3.** Length distribution of a) UWFs and b) HWFs, respectively.


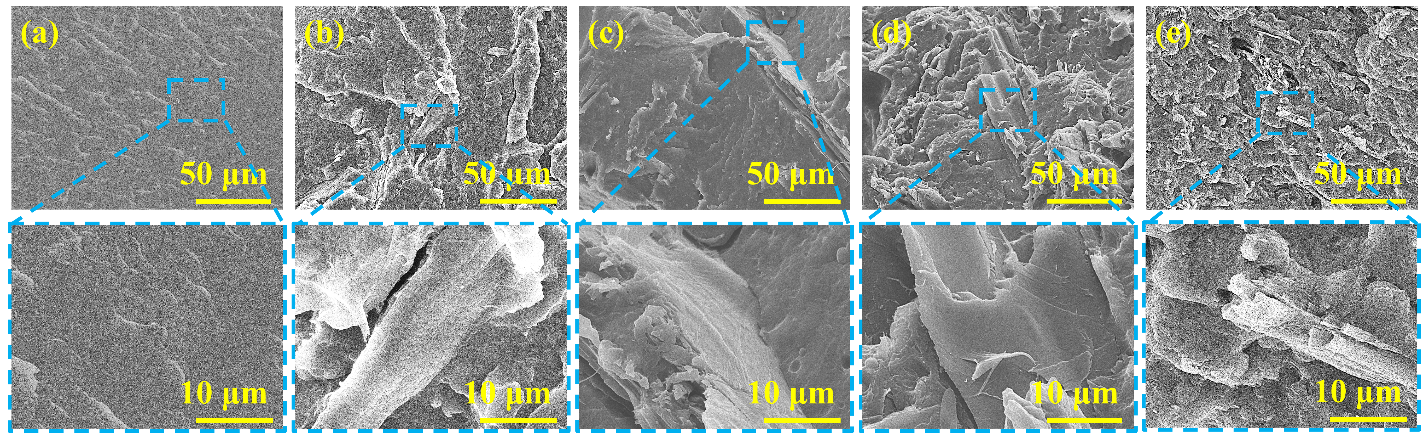


**Figure S4.** a) Fracture micro-morphology of polyamide matrix. b, d) and c, e) Fracture micro-morphology of UWPACs and HWPACs with 10% and 30% wood fiber content, respectively.


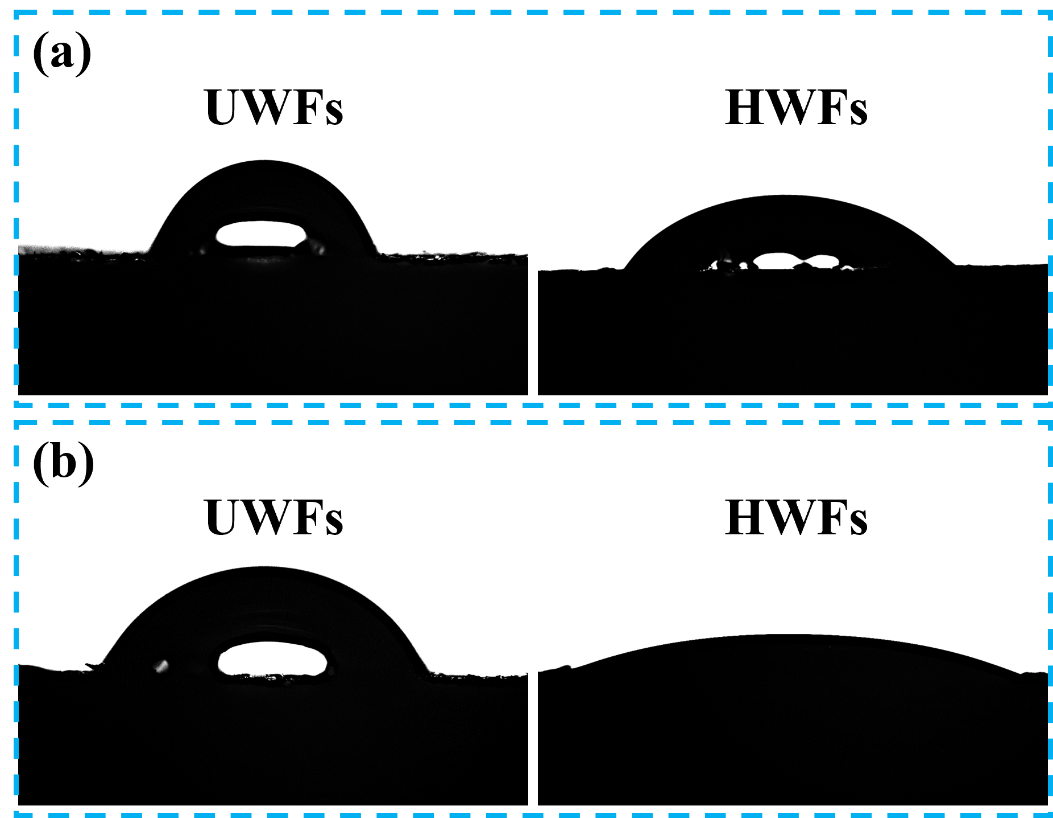


**Figure S5.** Contact angle images of UWFs and HWFs with a) water and b) formamide, respectively.


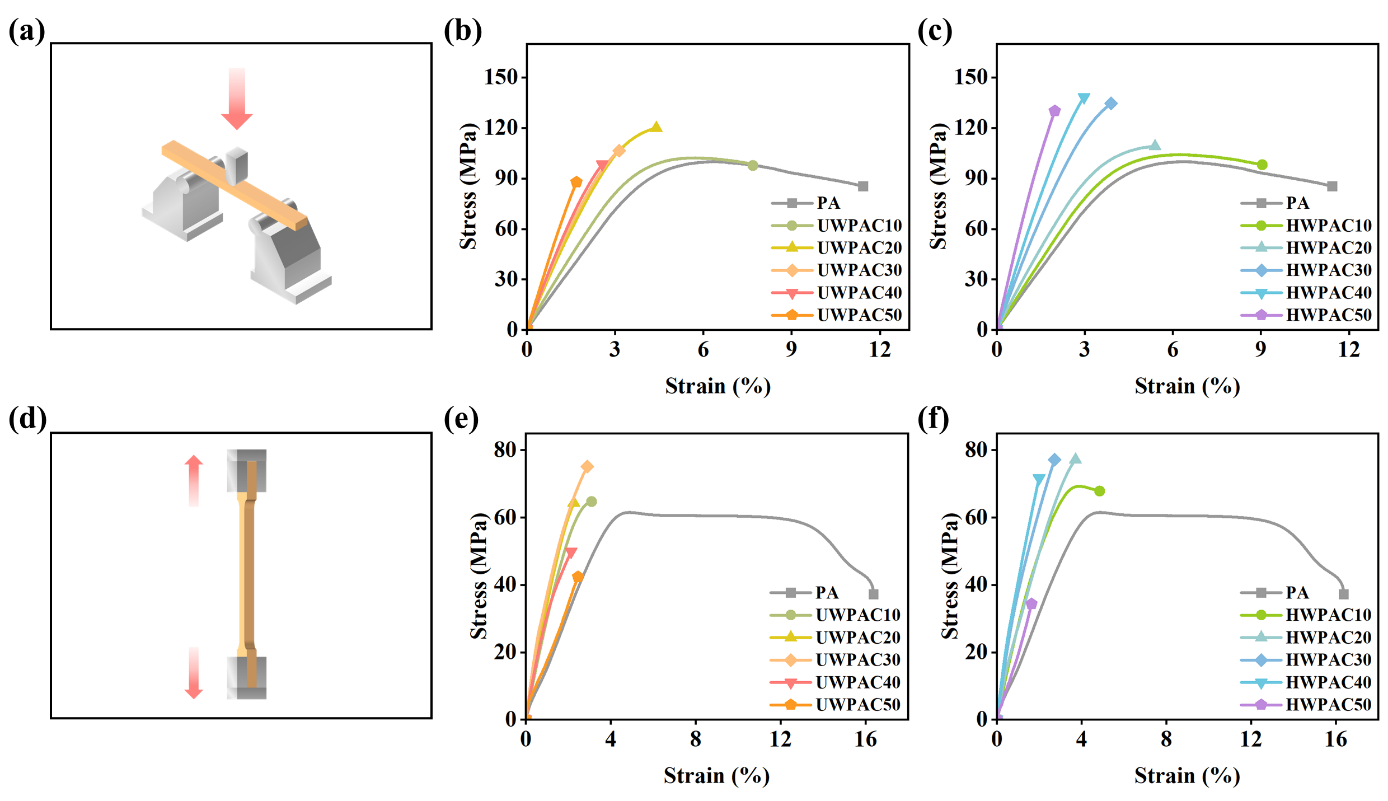


**Figure S6.** a, d) Schematic diagrams of flexural and tensile tests, respectively. b, c) Stress-strain curves of UWPACs and HWPACs in three-point flexural tests, respectively. e, f) Stress-strain curves of UWPACs and HWPACs in tensile tests, respectively.


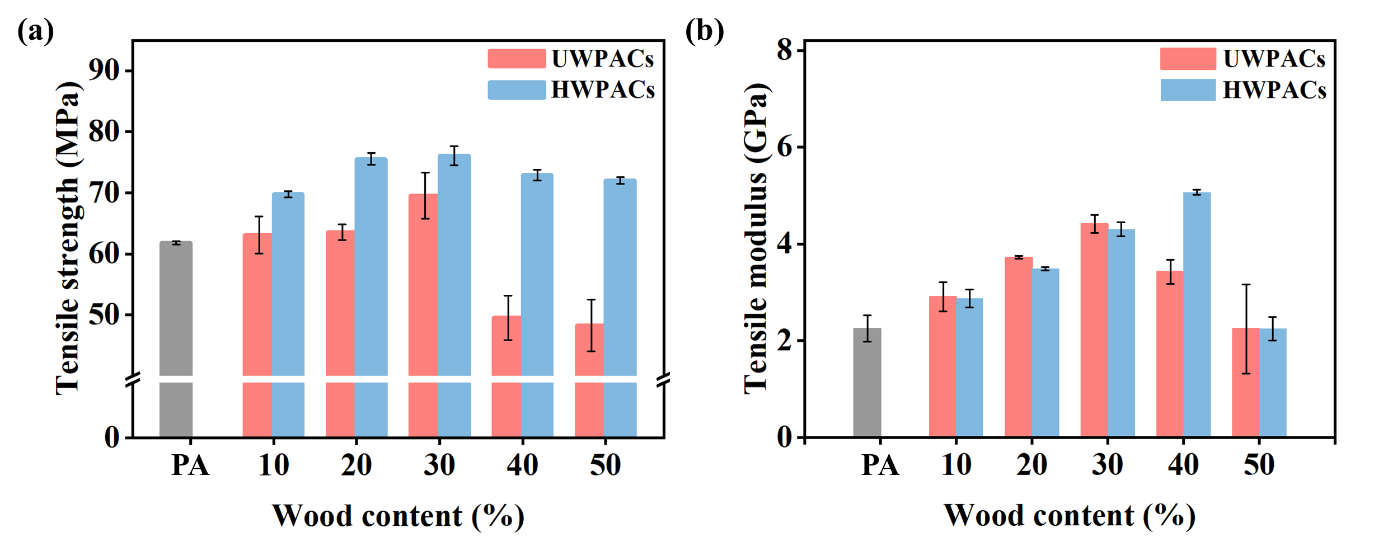


**Figure S7.** Tensile properties of UWPACs and HWPACs. a) tensile strength. b) tensile modulus.


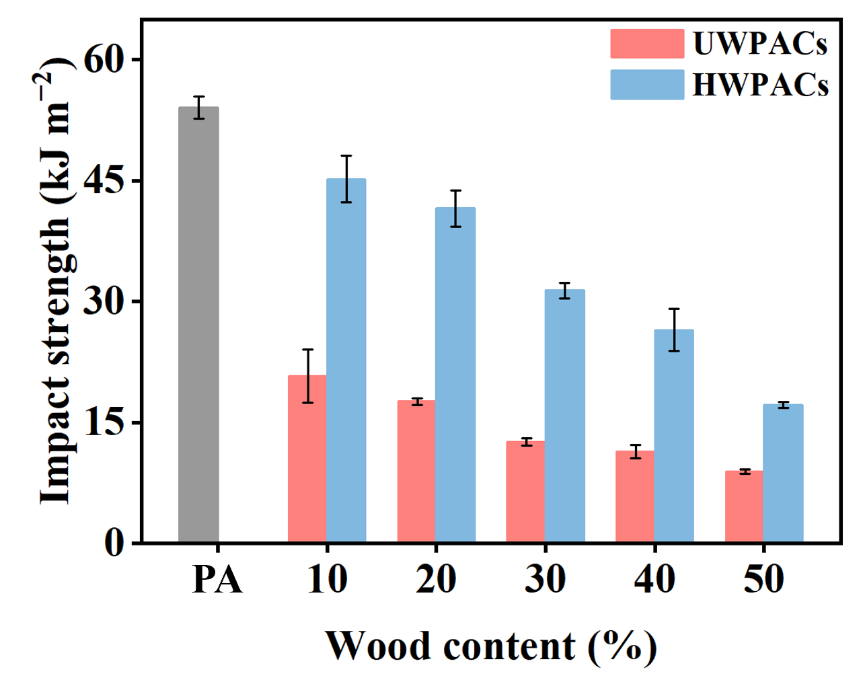


**Figure S8.** Impact strength of UWPACs and HWPACs.


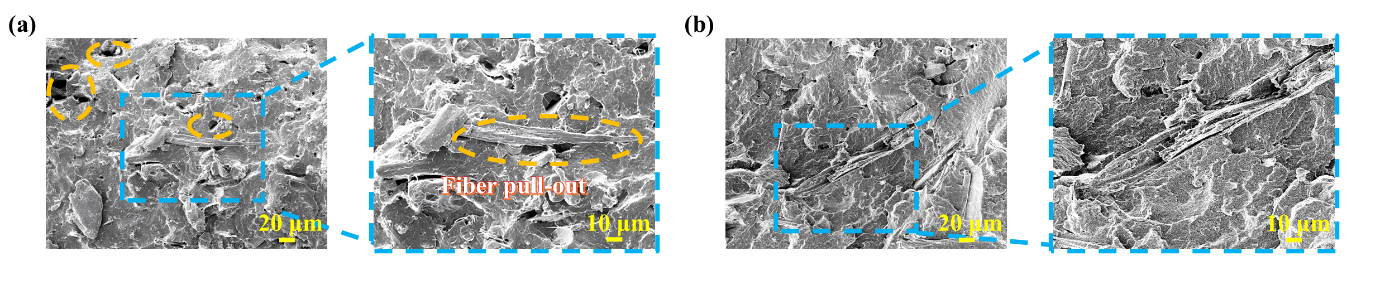


**Figure S9.** Tensile damage fractures of a) UWPACs and b) HWPACs, respectively.


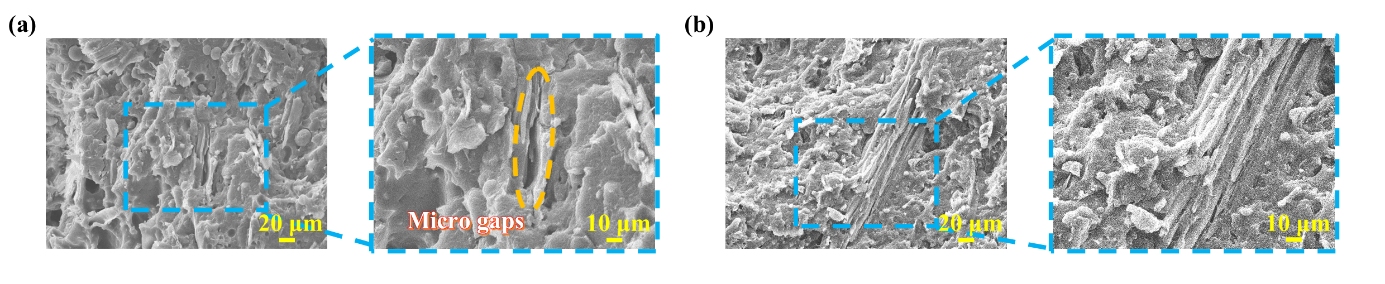


**Figure S10.** Impact damage fractures of a) UWPACs and b) HWPACs, respectively.


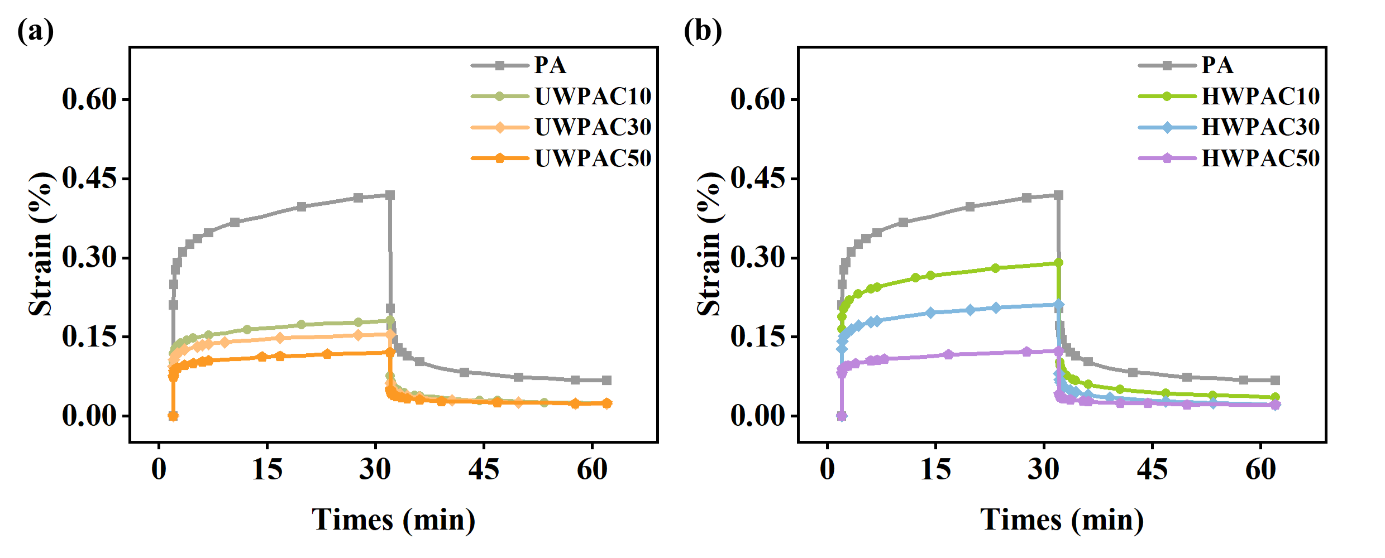


**Figure S11.** Creep and creep recovery of a) UWPACs and b) HWPACs at 30 °C, respectively.


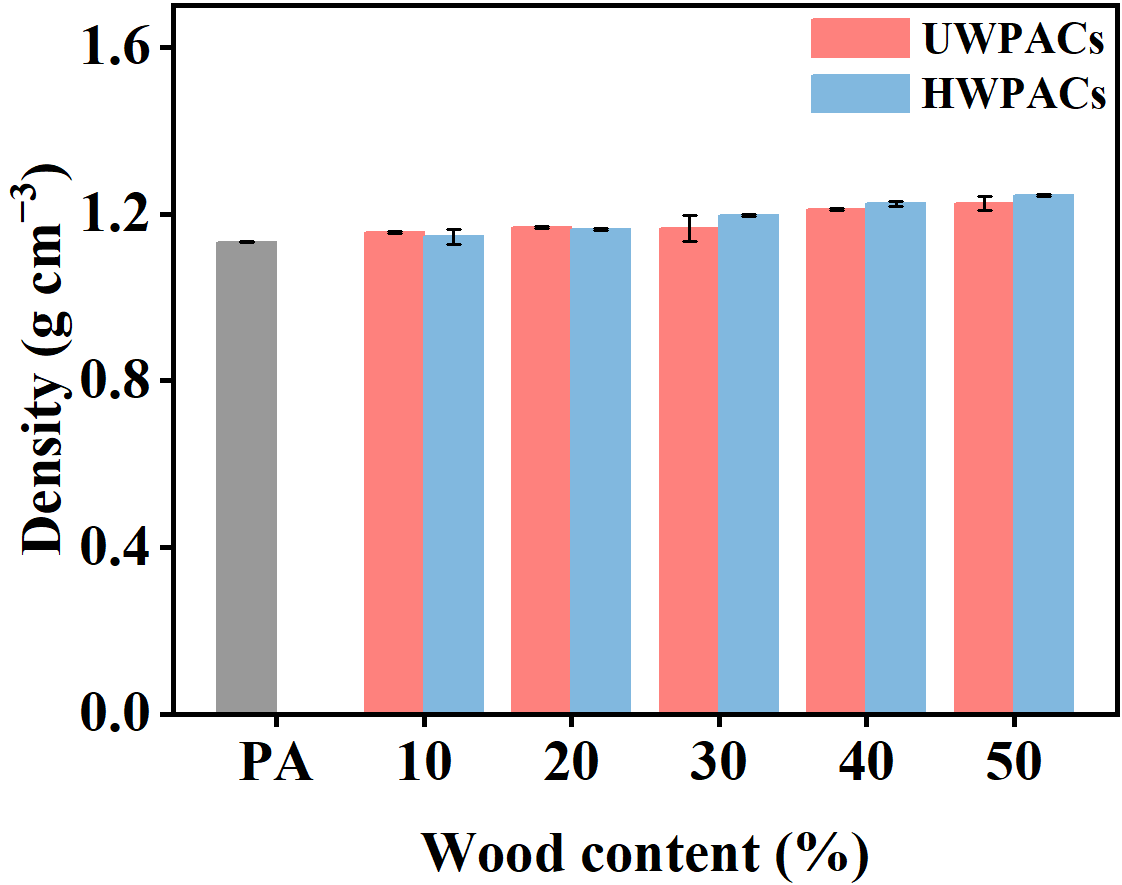


**Figure S12.** Density of UWPACs and HWPACs.


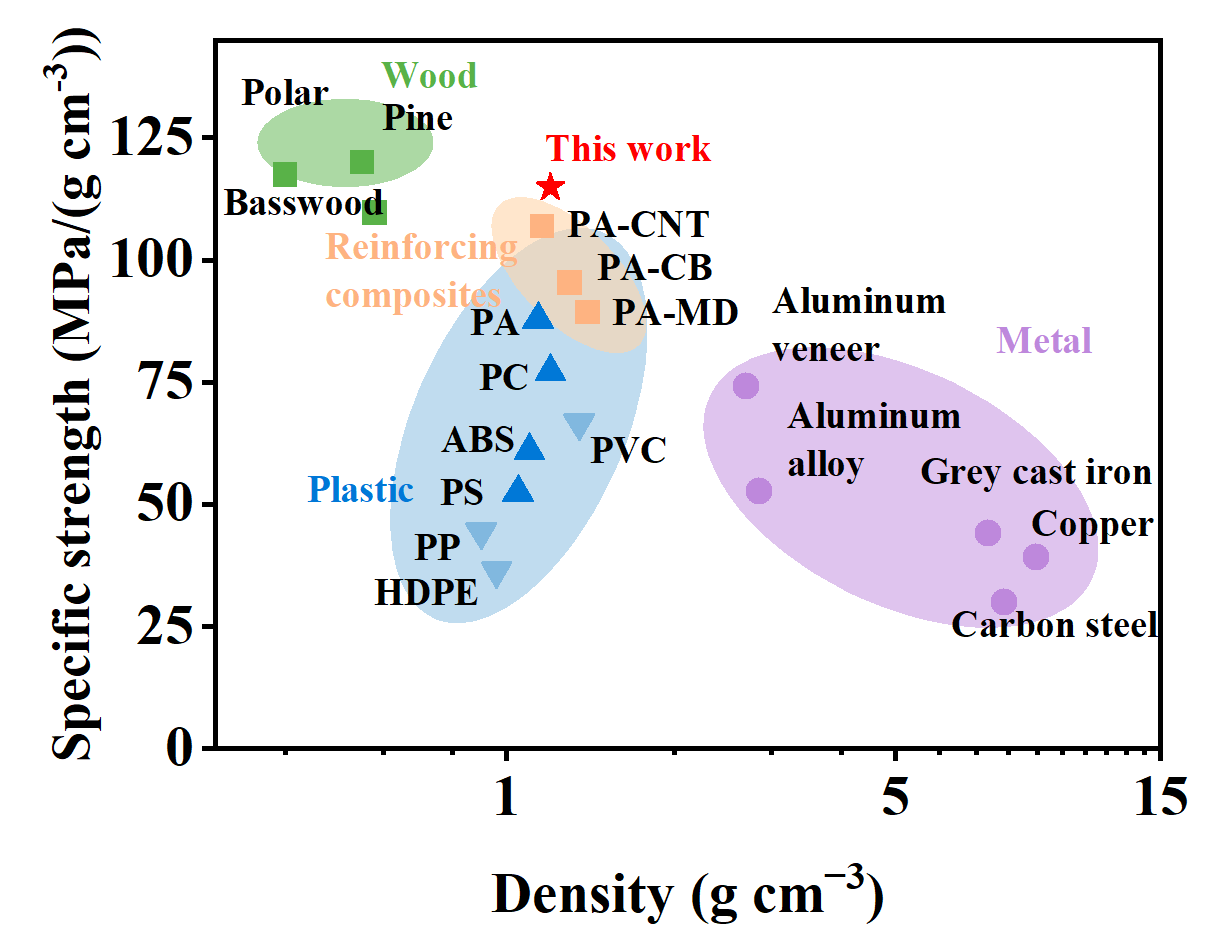


**Figure S13.** Material property map plotting specific strength against density of HWPACs and other common materials.


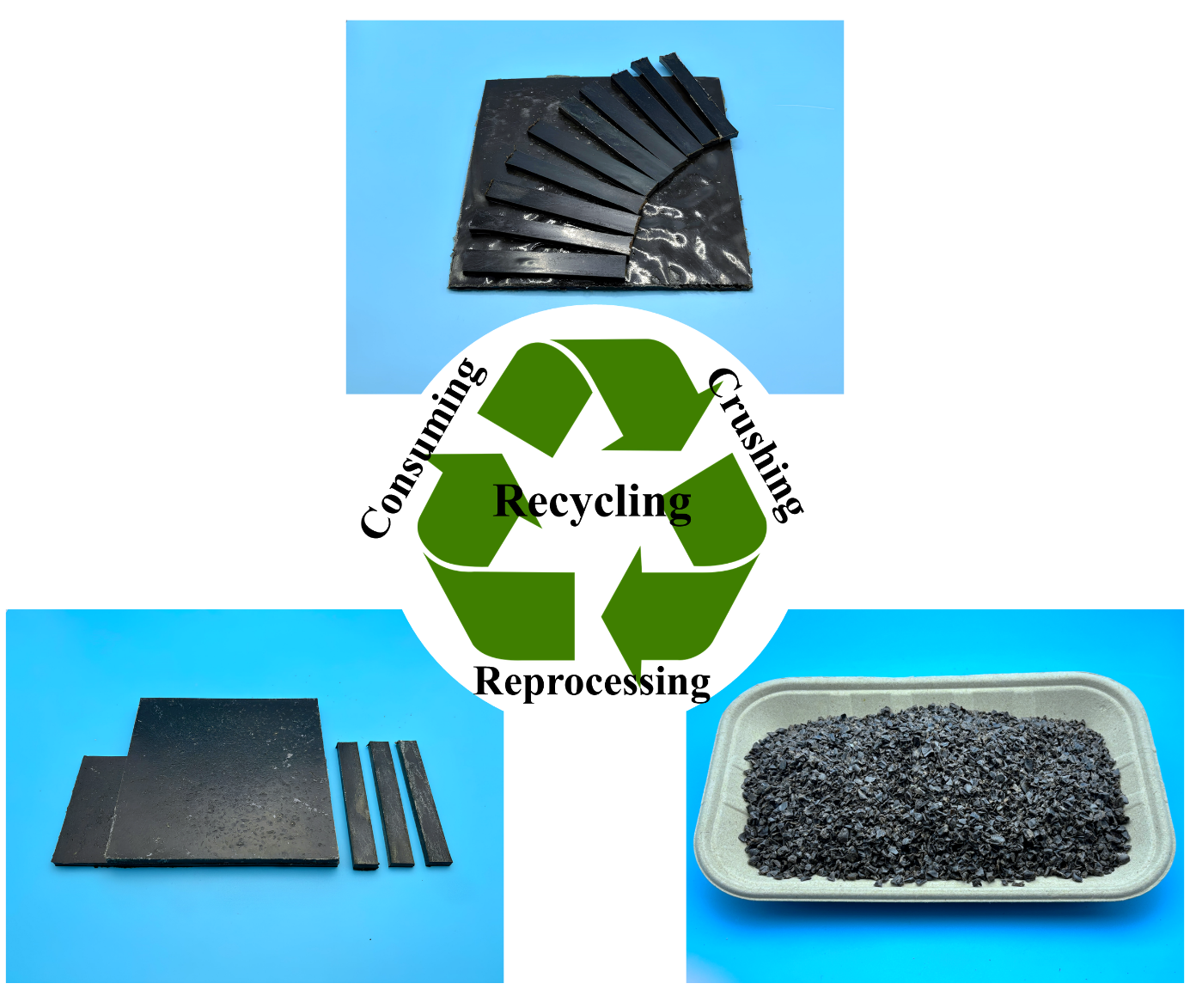


**Figure S14.** Schematic of the reprocessing cycle of HWPACs.

**
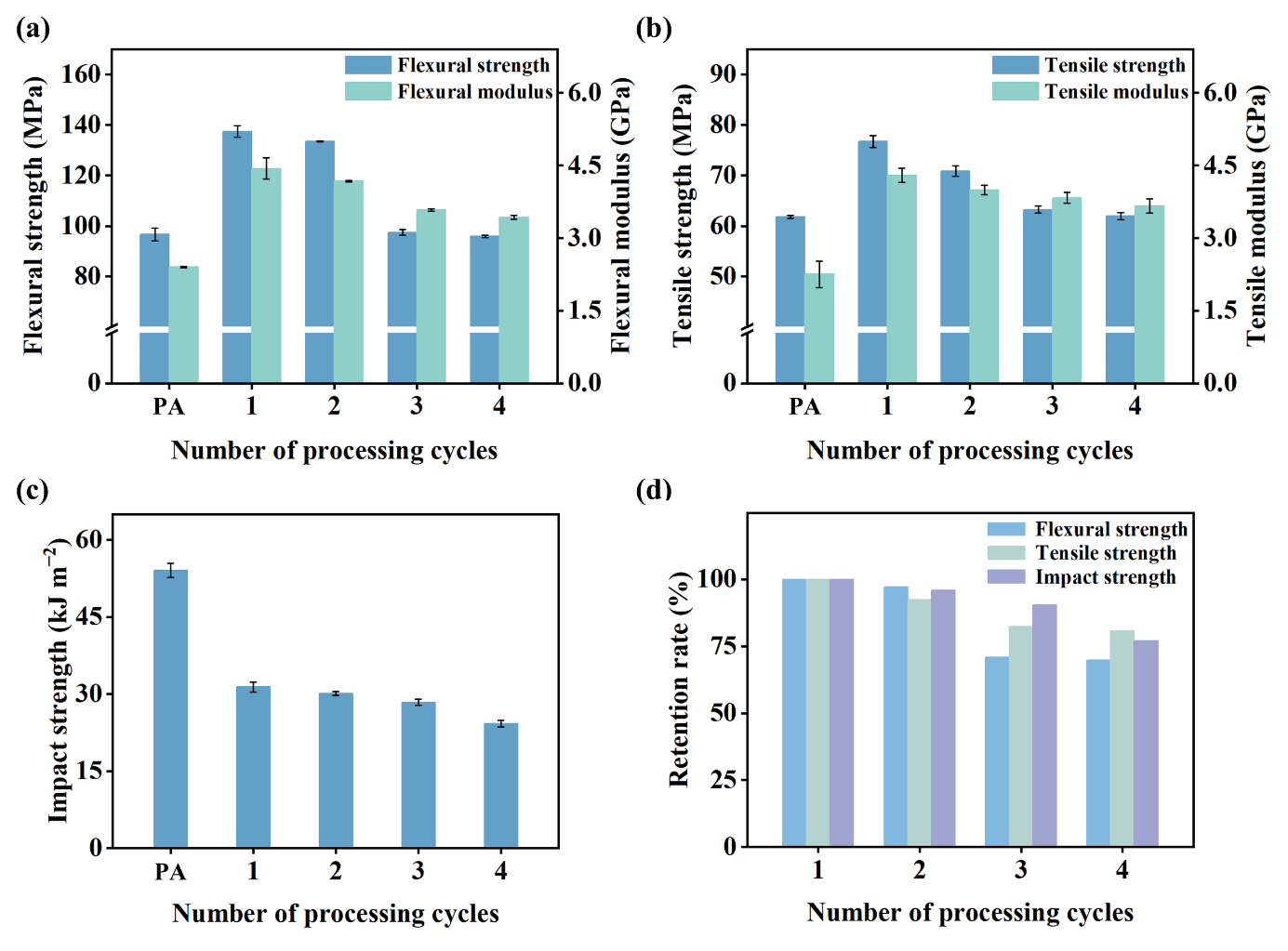
**

**Figure S15.** Mechanical properties of HWPAC30 with different number of processing cycles. a) Flexural properties. b) Tensile properties. c) Impact strength. d) Strength retention rate.


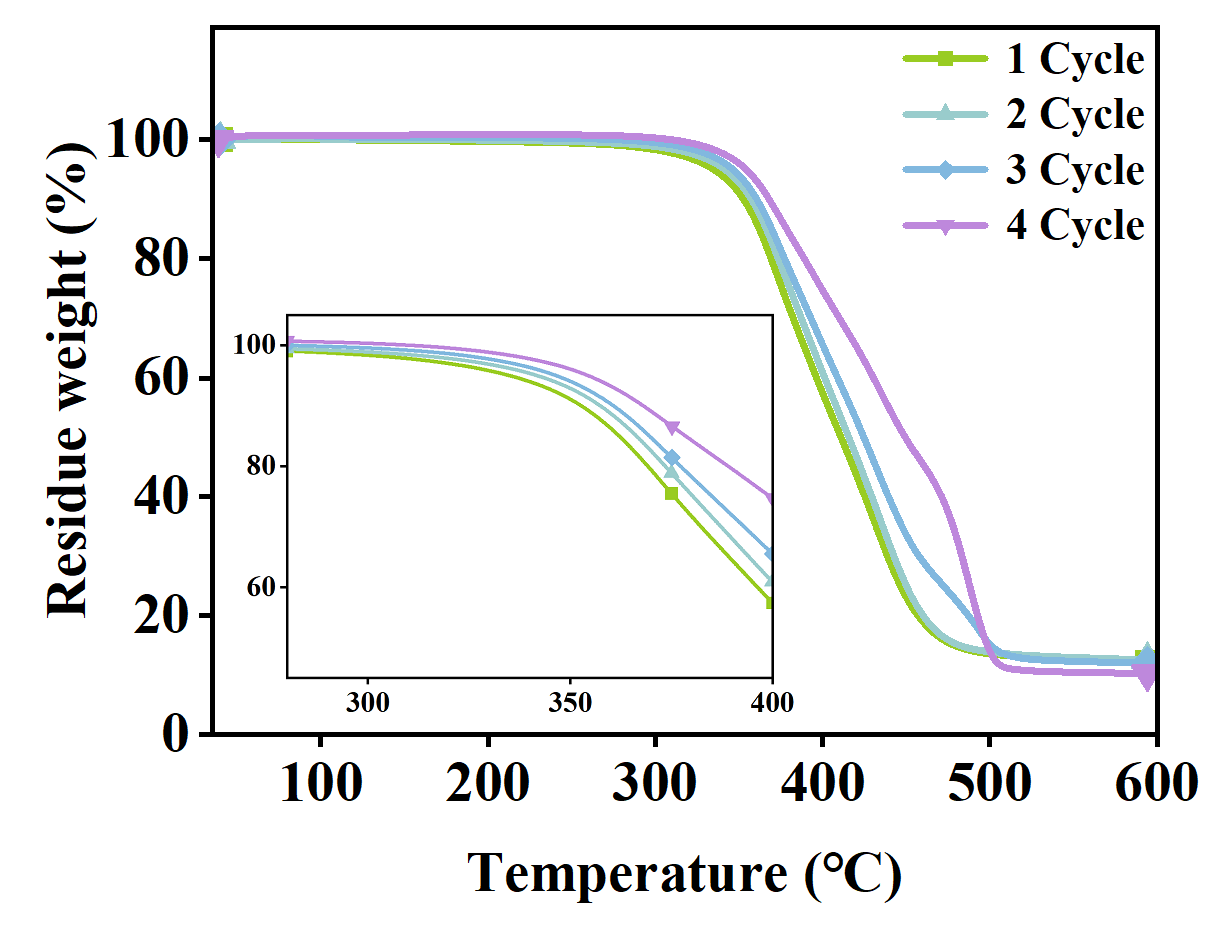


**Figure S16.** TGA curves of HWPAC30 with different number of processing cycles.


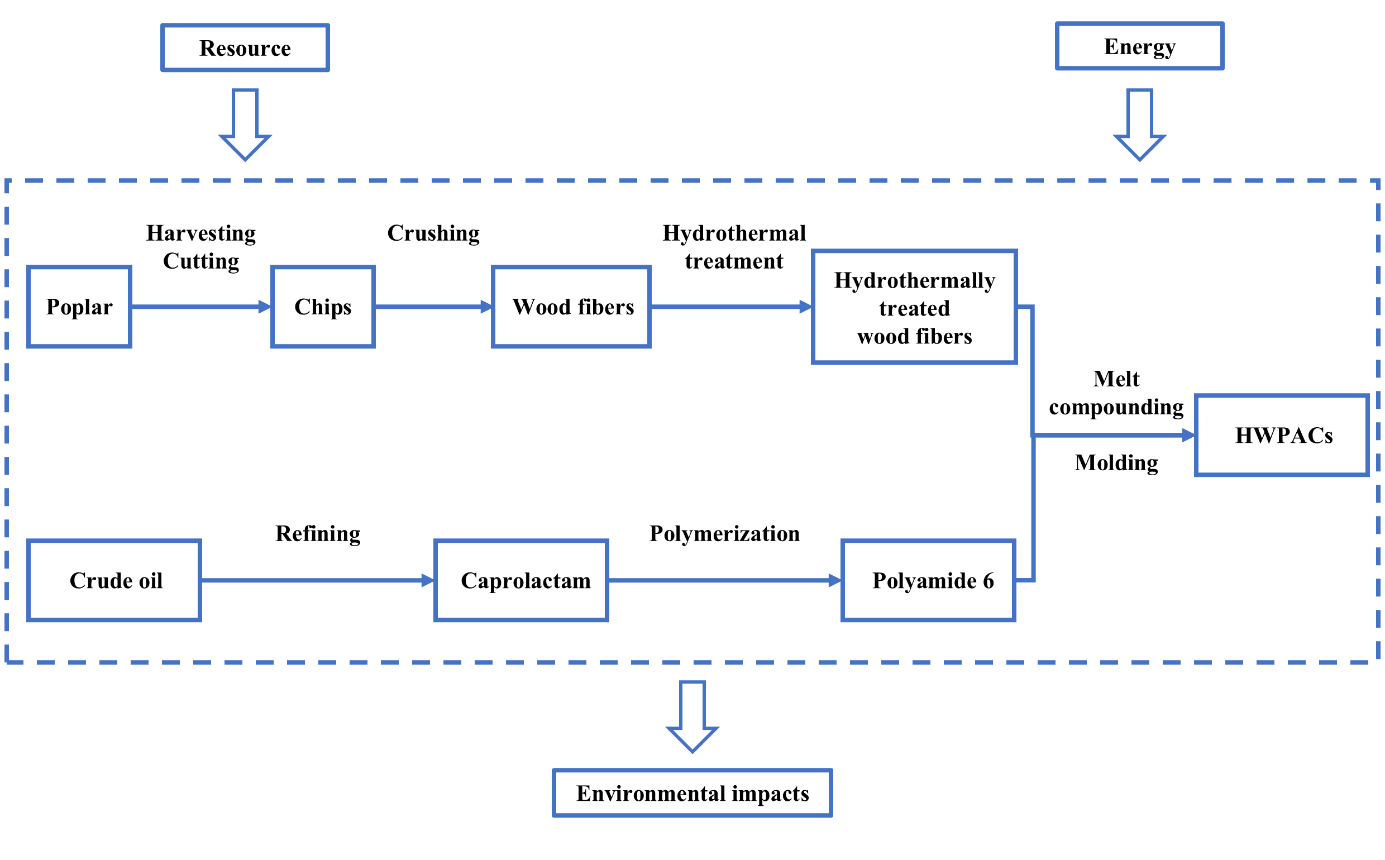


**Figure S17.** System boundary of life cycle assessment for the HWPACs.

**Table S1.** TGA parameters of the wood fibers and as-prepared composites.

| Samples | *T_o_* (°C) | *T_max_* (°C) | Char yield (%) |
| --- | --- | --- | --- |
| UWFs | 254.81 | 349.78 | 17.59 |
| HWFs | 282.30 | 364.75 | 17.34 |
| PA | 379.76 | 439.73 | 4.33 |
| UWPAC10 | 334.79 | 434.72 | 1.19 |
| UWPAC20 | 312.28 | 429.72 | 5.11 |
| UWPAC30 | 302.30 | 422.23 | 10.09 |
| UWPAC40 | 294.79 | 417.26 | 14.54 |
| UWPAC50 | 284.80 | 347.28 | 19.31 |
| HWPAC10 | 349.77 | 437.21 | 6.81 |
| HWPAC20 | 334.78 | 427.22 | 9.00 |
| HWPAC30 | 322.29 | 419.73 | 11.34 |
| HWPAC40 | 314.79 | 357.26 | 14.47 |
| HWPAC50 | 304.80 | 352.27 | 20.85 |

**Table S2.** Morphological characteristics of UWFs and HWFs.

| Samples | Fiber length (mm) | Fiber width (mm) | Aspect ratio |
| --- | --- | --- | --- |
| UWFs | 1.41 ± 0.48 | 0.16 ± 0.04 | 8.81 |
| HWFs | 0.97 ± 0.41 | 0.12 ± 0.04 | 8.26 |

**Table S3.** The flexural strength and modulus of this work and other reported biomass/polyamide composites.

| Samples | Flexural strength (MPa) | Flexural modulus (GPa) | Reference |
| --- | --- | --- | --- |
| HWPAC40 | 139.45 | 5.57 | This work |
| BWPAC | 65.40 | 3.90 | [1] |
| PA11+60SGW | 102.70 | 4.10 | [2] |
| LPA6/WFC | 85.32 | 3.64 | [3] |
| PA-20WF | 77.30 | 2.19 | [4] |
| HDPE/PA6/WF-HTV composites | 44.64 | 4.02 | [5] |
| WF10CF20 | 141.95 | 4.31 | [6] |
| BC-2 | 105.47 | 2.71 | [7] |
| RN-600 | 121.66 | 3.70 | [8] |
| PA6/BioC30 | 93.38 | 2.84 | [9] |
| lignin-based CF reinforced PA66 | 66MPa | 3.4GPa | [10] |

**Table S4.** Comparison of the onset thermal degradation temperature (*T_o_*) of this work and other reported biomass/polymer composites.

| Samples | *T_o_* (°C) | Reference |
| --- | --- | --- |
| HWPACs | 322.29 | This work |
| BWPAC | 301.06 | [1] |
| LPA6/WFC | 141.35 | [3] |
| WF/PVCMZ | 215.30 | [11] |
| WPCC30 | 295.02 | [12] |
| LLDPE/WF-S3M | 274.00 | [13] |
| WF/PPMZ | 267.80 | [14] |

**Table S5.** DSC parameters for UWPACs and HWPACs.

| Samples | PA6 | UWPAC10 | UWPAC20 | UWPAC30 | UWPAC40 | UWPAC50 |
| --- | --- | --- | --- | --- | --- | --- |
| *T_m_* (°C) | 221.22 | 219.94 | 219.96 | 218.98 | 217.05 | 215.12 |
| *X_C_* (%) | 19.48 | 18.44 | 18.74 | 20.37 | 21.08 | 19.45 |
| Samples | – | HWPAC10 | HWPAC20 | HWPAC30 | HWPAC40 | HWPAC50 |
| *T_m_* (°C) | – | 219.87 | 219.34 | 219.10 | 218.31 | 216.77 |
| *X_C_* (%) | – | 19.69 | 19.78 | 20.74 | 22.72 | 21.33 |

**Table S6.** Input and output of the HWPACs production process presented in this work.

| Infrastructure Processes^a)^ | Unit | Quantity |
| --- | --- | --- |
| Inputs from technosphere |  |  |
| Electricity^b)^, medium voltage, CN | kWh | 2028.87 |
| Wood fibers by-product | kg | 679.44 |
| Polyamide 6, production | kg | 664.82 |
| Inputs from nature |  |  |
| Water | kg | 3397.21 |
| Outputs to technosphere: Products |  |  |
| HWPACs | kg | 1000.00 |
| Outputs to nature |  |  |
| Water (evapotranspiration) | kg | 49.25 |
| Waste water | kg | 186.98 |
| VOC | kg | 0.02 |
| Carbon dioxide | kg | 738.00 |
| Carbon monoxide | kg | 1.20 |
| Methane | kg | 1.75 |
| Nitrogen oxides | kg | 7.19 |
| Sulfur oxides | kg | 4.47 |
| Ammonia | kg | 0.22 |

^a)^(The input and output of the production process for 1000 kg HWPACs were calculated according to the scope of this study. The data used were based on published literature^[15]^ or professional judgment.)

^b)^(The preparation processes of HWPACs (drying, extrusion compounding and injection molding) are powered using electricity.)

**Table S7.** The environmental impacts of HWPACs, PA6 and other commercial traditional fiber reinforced polyamide composites.

| Impact category | Unit | HWPACs | PA6 | GF-PA | CF-PA | CB-PA |
| --- | --- | --- | --- | --- | --- | --- |
| Abiotic depletion | kg Sb eq | 4.54 × 10^−5^ | 7.05 × 10^−5^ | 6.17 × 10^−5^ | 7.47 × 10^−5^ | 7.12 × 10^−5^ |
| Global warming (GWP100a) | kg CO_2_ eq | 8.92 | 14.22 | 11.85 | 21.59 | 14.24 |
| Ozone layer depletion (ODP) | kg CFC-11 eq | 1.47 × 10^−8^ | 1.67 × 10^−8^ | 6.69 × 10^−8^ | 5.60 × 10^−8^ | 7.80 × 10^−8^ |
| Human toxicity | kg 1,4-DB eq | 1.01 | 1.14 | 2.34 | 3.62 | 1.30 |
| Fresh water aquatic ecotoxicity | kg 1,4-DB eq | 0.67 | 0.69 | 1.22 | 2.53 | 0.81 |
| Marine aquatic ecotoxicity | kg 1,4-DB eq | 3.57 × 10^3^ | 3.51 × 10^3^ | 5.52 × 10^3^ | 14.95 × 10^3^ | 3.93 × 10^3^ |
| Terrestrial ecotoxicity | kg 1,4-DB eq | 4.11 × 10^−3^ | 4.29 × 10^−3^ | 6.00 × 10^−3^ | 11.36 × 10^−3^ | 4.72 × 10^−3^ |
| Photochemical oxidation | kg C_2_H_4_ eq | 1.29 × 10^−3^ | 1.86 × 10^−3^ | 1.61 × 10^−3^ | 3.09 × 10^−3^ | 1.89 × 10^−3^ |
| Acidification | kg SO_2_ eq | 3.28 × 10^−2^ | 5.08 × 10^−2^ | 4.70 × 10^−2^ | 8.38 × 10^−2^ | 5.16 × 10^−2^ |
| Eutrophication | kg PO_4_ eq | 7.11 × 10^−3^ | 11.18 × 10^−3^ | 10.51 × 10^−3^ | 18.65 × 10^−3^ | 11.32 × 10^−3^ |

**Table S8.** Results of the contribution analysis.

|  |  | Hydrothermal treatment | | Melt compounding | | | Injection molding | |
| --- | --- | --- | --- | --- | --- | --- | --- | --- |
| Impact category | Total | Wood fibers | Electricity | Polyamide 6 | Electricity | Emission | Electricity | Emission |
| Abiotic depletion | 100% | 0.05% | 0.46% | 95.70% | 1.10% | 0.00% | 2.68% | 0.00% |
| Global warming (GWP100a) | 100% | 0.09% | 2.52% | 68.05% | 5.98% | 1.76% | 14.53% | 7.06% |
| Ozone layer depletion (ODP) | 100% | 0.27% | 7.28% | 33.15% | 17.29% | 0.00% | 42.00% | 0.00% |
| Human toxicity | 100% | 0.33% | 7.69% | 28.50% | 18.26% | 0.17% | 44.36% | 0.68% |
| Fresh water aquatic ecotoxicity | 100% | 0.53% | 9.28% | 14.60% | 22.05% | 0.00% | 53.55% | 0.00% |
| Marine aquatic ecotoxicity | 100% | 0.37% | 9.90% | 9.05% | 23.53% | 0.00% | 57.15% | 0.00% |
| Terrestrial ecotoxicity | 100% | 0.73% | 8.81% | 18.68% | 20.93% | 0.00% | 50.84% | 0.00% |
| Photochemical oxidation | 100% | 0.10% | 2.88% | 70.20% | 6.85% | 0.66% | 16.64% | 2.66% |
| Acidification | 100% | 0.11% | 3.05% | 60.00% | 7.24% | 2.38% | 17.58% | 9.64% |
| Eutrophication | 100% | 0.11% | 3.09% | 57.42% | 7.33% | 2.83% | 17.81% | 11.41% |

**Table S9.** Formulation of as-prepared wood fiber/PA 6 composites.

| Samples | Polyamide 6 (wt %) | Untreated wood fibers (wt %) | Hydrothermally treated wood fibers (wt %) |
| --- | --- | --- | --- |
| PA | 100 | – | – |
| UWPAC10 | 90 | 10 | – |
| UWPAC20 | 80 | 20 | – |
| UWPAC30 | 70 | 30 | – |
| UWPAC40 | 60 | 40 | – |
| UWPAC50 | 50 | 50 | – |
| HWPAC10 | 90 | – | 10 |
| HWPAC20 | 80 | – | 20 |
| HWPAC30 | 70 | – | 30 |
| HWPAC40 | 60 | – | 40 |
| HWPAC50 | 50 | – | 50 |

References

[1] J. Zhang, A. Koubaa, D. Xing, W. Liu, Q. Wang, X. Wang, H. Wang, *Mater. Des.* **2020**, 191, 108589.

[2] H. Oliver-Ortega, F. Julian, F. X. Espinach, Q. Tarrés, M. Ardanuy, P. Mutjé, *J. Cleaner Prod.* **2019**, 226, 64.

[3] S. Xu, L. Sun, J. He, H. Han, H. Wang, Y. Fang, Q. Wang, *Polym. Compos.* **2018**, 39, E1574.

[4] B. E. Hirin, H. Yrür, F. J. B. Mengelolu, *BioResources* **2020**, 16, 655.

[5] K. Xu, Z. Zheng, G. Du, Y. Zhang, Z. Wang, T. Zhong, L. Xie, S. Wang, *J. Appl. Polym. Sci.* **2019**, 136, 47984.

[6] S. Armioun, S. Panthapulakkal, J. Scheel, J. Tjong, M. Sain, *J. Appl. Polym. Sci.* **2016**, 133, 43595.

[7] E. O. Ogunsona, M. Misra, A. K. Mohanty, *J. Appl. Polym. Sci.* **2017**, 134, 44221.

[8] Z. B. Varsally, N. Tripathi, H. Weldekidan, A. Rodriguez-Uribe, O. Das, A. K. Mohanty, M. Misra, *Compos. Part C-Open* **2023**, 12, 100376.

[9] J. Andrzejewski, J. Aniśko, J. Szulc, *Compos. Part A-Appl S.* **2022**, 152, 106715.

[10] A. Beaucamp, M. Muddasar, M. Culebras, M. N. Collins, *Compos. Commun.* **2024**, 45, 101782.

[11] R. Yang, Y. Fang, J. Zhu, G. Ren, B. Lin, F. Wang, R. Ou, Q. Wang, Y. Song, *Constr. Build. Mater.* **2024**, 420, 135522.

[12] J. Zhang, A. Koubaa, D. Xing, W. Liu, H. Wang, X. Wang, Q. Wang, *Compos. Part A-Appl S.* **2020**, 138, 106068.

[13] S. Yang, B. Wei, Q. Wang, *Compos. Part B-Eng.* **2020**, 200, 108347.

[14] R. Yang, Y. Fang, Y. Fu, J. Zhu, G. Ren, X. Bai, Y. Song, W. Wang, Q. Wang, *ACS Sustainable Chem. Eng.* **2024**, 12, 9101.

[15] T. Qiang, D. Yu, A. Zhang, H. Gao, Z. Li, Z. Liu, W. Chen, Z. Han, *J. Cleaner Prod.* **2014**, 66, 139.
